# Identification of key genes responsible for green and white colored spathes in *Anthurium andraeanum* (Hort.)

**DOI:** 10.3389/fpls.2023.1208226

**Published:** 2023-09-06

**Authors:** Jieni Li, Quanya Tan, Maosheng Yi, Zhengnan Yu, Qing Xia, Lu Zheng, Jianjun Chen, Xiaoyun Zhou, Xiang-Qian Zhang, He-Rong Guo

**Affiliations:** ^1^ College of Forestry and Landscape Architecture, South China Agricultural University, Guangzhou, China; ^2^ Guangdong Provincial Key Laboratory of Plant Molecular Breeding, South China Agricultural University, Guangzhou, China; ^3^ Guangzhou Flower Research Center, Guangzhou, China; ^4^ Mid-Florida Research and Education Center, Environmental Horticulture Department, Institute of Food and Agricultural Sciences, University of Florida, Apopka, FL, United States

**Keywords:** anthocyanin, *Anthurium andraeanum*, spathe color, flavonoid, chlorophyll, *MYB* transcription factor

## Abstract

Modern anthuriums, *Anthurium andraeanum* (Hort.) are among the most popular flowering plants and widely used for interior decoration. Their popularity is largely attributed to the exotic spathes with different colors. Previous studies have reported color development in red spathe cultivars, but limited information is available on key genes regulating white and green colored spathes. This study analyzed anthocyanin, chlorophyll, and carotenoid contents as well as transcript differences in spathes of eight cultivars that differed in spathe colors ranging from red to white and green. Results showed that increased expression of a transcription factor *AaMYB2* was associated with elevated levels of anthocyanin in spathes, but decreased expression of *AaMYB2* and increased expression of *AaLAR* (leucoanthocyanidin reductase) and *AaANR* (anthocyanidin reductase) were accompanied with the accumulation of colorless proanthocyanidin, thus the white spathe. As to the green colored spathe, chlorophyll content in the green spathe cultivar was substantially higher than the other cultivars. Correspondingly, transcripts of chlorophyll biosynthesis-related genes *AaHemB* (porphobilinogen synthase) and *AaPor* (protochlorophyllide oxidoreductase) were highly upregulated but almost undetectable in white and red spathes. The increased expression of *AaHemB* and *AaPor* was correlated with the expression of transcription factor *AaMYB124*. Subsequently, qRT-PCR analysis confirmed their expression levels in nine additional cultivars with red, white, and green spathes. A working model for the formation of white and green spathes was proposed. White colored spathes are likely due to the decreased expression of *AaMYB2* which results in increased expression of *AaLAR* and *AaANR*, and the green spathes are attributed to *AaMYB124* enhanced expression of *AaHemB* and *AaPor*. Further research is warranted to test this working model.

## Introduction

*Anthurium* Schott is the largest genus in the family Araceae and comprises more than 1,000 species (Croat, 1992). Among them, *A. andraeanum* Linden ex Andre and *A. scherzerianum* Schott were produced as cut flower and potted flowering plants, respectively (Kamemoto and Kuehnle, 1996; Anthura, 2002). With the efforts in anthurium breeding, increasing numbers of anthurium cultivars have been developed through the hybridization of *A. andraeanum* with other species or cultivars (Marco van Herk et al., 1998; Anthura, 2002). These commercial cultivars are complex interspecific hybrids (Elibox and Umaharan, 2008) and are collectively referred to as *A. andraeanum* (Hort.) or modern anthuriums to distinguish them from *A. andraeanum* Linden ex Andre (Kamemoto and Kuehnle, 1996).

*Anthurium andraeanum* (Hort.) cultivars have some distinct characteristics: (1) Spathes vary greatly in color, size, shape, and texture (Elibox and Umaharan, 2008); (2) they have more compact growth forms and can be used as either cut flower or potted flowering plants (Chen et al., 1999); and (3) spathes have extended longevity (Kamemoto and Kuehnle, 1996; Chen et al., 2002). As a result, these cultivars have quickly gained popularity in the world floral market. The turnover of potted anthurium was €9.94 million in 1997 and increased to €23.22 million in 2001 in Dutch auctions (Anthura, 2002), and subsequently the turnover increased to €50 million (Rikken, 2010). In China, the wholesale value of anthuriums ranked second after orchids, and more than 20 million potted anthuriums were sold in 2013, of which 6.5 million were sold during the Chinese New Year (Hua, 2014).

The increasing popularity is mainly attributed to their exotic shape and colorful spathes (Henny and Chen, 2003). The spathe is actually a modified leaf that initially covers a cylindrical shaped spadix on which both male and female flowers are found. The primary colors of spathes are red including bright red, dark red, pink, orange, brown, and coral as well as white. To increase colorations, cultivars with green, brown, and bi-color spathes were developed (Kamemoto and Kuehnle, 1996). Since color is by far the most important characteristic of ornamental plants (Halloran and Kuehnle, 1998; Chen, 2021), there is a continuous effort to develop more colorful spathes in anthurium breeding using genetic manipulation (Gopaulchan et al., 2013; Teixeira da Silva et al., 2015; Li et al., 2016; Li et al., 2019).

Flower color is primarily determined by pigments (Clegg and Durbin, 2000). There are four major pigments in plants: anthocyanins, betalains, carotenoids, and chlorophyll. Among them, anthocyanins, carotenoids, and chlorophyll are implicated in anthurium spathe color, and their concentrations and ratios define the spathe color and its intensity (Lin et al., 2022). Betalains are restricted to the core Caryophyllales and do not occur in other plants including anthurium (Timoneda et al., 2019).

Anthocyanins are the largest group of pigments ranging from pink, red, purple to blue, which is synthesized through the flavonoid pathway (Pucker and Selmar, 2022). Cinnamate-4-hydroxylase (C4H) plays a key role in flavanol accumulation during the early stage (Mizutani et al., 1993; Ren et al., 2019). Chalcone synthase (CHS) converts 4-coumaroyl-CoA and malonyl-CoA to naringenin chalcone (Feinbaum and Ausubel, 1988; Franken et al., 1991). Then naringenin chalcone is isomerized to naringenin through chalcone isomerase (CHI) (Grotewold and Peterson, 1994). The formation of dihydrokaempferol and dihydroquercetin from naringenin is catalyzed by flavanone 3-hydroxylase (F3H) and flavonoid 3′-hydroxylase (F3’H), respectively (Sparvoli et al., 1994; Brugliera et al., 1999). The conversion of dihydroflavonols into leucoanthocyanidins is catalyzed by dihydroflavonol 4-reductase (DFR) (Helariutta et al., 1993). Leucoanthocyanidins are channeled into anthocyanidins by anthocyanidin synthase (ANS) (Saito et al., 1999); and meanwhile leucoanthocyanidin reductase (LAR) converts leucoanthocyanidins to catechin (Tanner et al., 2003; Akagi et al., 2009). Anthocyanidin then may be converted to epicatechin by anthocyanidin reductase (ANR) (Xie et al., 2004; Akagi et al., 2009). Both catechin and epicatechin are colorless. Colored anthocyanidins were produced by flavonoid 3-O-glucosyl transferase (UFGT) (Boss et al., 1996; Fan et al., 2022). Anthocyanin O-methyltransferase (AOMT) is responsible for the methylation of cyanidin glycosides and plays an important role in purple coloration (Du et al., 2015). Flavonoids are synthesized in the cytosol and then transported to the vacuole. Glutathione S-transferase (GST) participates in flavonoid transport (Mueller et al., 2000; Kitamura et al., 2012; Sun et al., 2012).

Chlorophylls and carotenoids are photosynthetic pigments, which are biosynthesized in plastids from metabolic precursors derived from the methylerythritol 4-phosphate (MEP) pathway (Rodriguez-Concepcion and Boronat, 2002). Chlorophylls are green pigments. A series of important enzymes are involved in chlorophyll metabolism, such as porphobilinogen synthase (HemB) and protochlorophyllide reductase (Por) (Zhou et al., 2022). Carotenoids range from colorless to yellow, orange, and red; and a large number of genes participates in carotenoid biosynthesis, including phytoene desaturase gene (*PDS*), β-ring carotene hydroxylase gene (*CrtR-b*), zeaxanthin epoxidase gene (*ZEP*), and lutein deficient 5 gene (*LUT5*) (Nisar et al., 2015).

Transcription factors (TF), like *R2R3-MYB* and members of the basic helix-loop-helix (*bHLH*) family, are the most important classes of transcriptional regulators controlling secondary metabolism, developmental processes, and signal transduction (Yan et al., 2021). Anthocyanin and proanthocyanidin are secondary metabolites. The *MYB*-TF family has been found to act favorably in regulation of the pigment pathway by binding directly to the promoter of pigment biosynthesis genes (Aharoni et al., 2001; Ithal and Reddy, 2004; Jiang et al., 2021; Yang et al., 2022). Flavonoid pathway is also regulated by a combination model of transcription factors, including members of *MYB*, *bHLH*, and WD-repeat (*WDR*) families (Gonzalez et al., 2008; Petroni and Tonelli, 2011; Li, 2014). In *Arabidopsis*, *TT2* (*MYB123*), *TT8* (*bHLH042*), and *TTG1* (*WDR*) are shown to participate in the control of proanthocyanidin biosynthesis (Baudry et al., 2004; Baudry et al., 2006; Peng et al., 2020). Overexpression of *MYB75/PAP1* and *MYB90/PAP2*, whose sequences are similar to *MYB113* and *MYB114*, respectively, results in the accumulation of anthocyanin (Borevitz et al., 2000; Gonzalez et al., 2008). *MYB11*, *MYB12*, and *MYB111* control flavonol accumulation in different parts of the *Arabidopsis* seedling (Mehrtens et al., 2005; Stracke et al., 2007). *MYB* TFs activate or suppress anthocyanin biosynthesis genes, which either improve or inhibit anthocyanin accumulation (Li et al., 2022). In *Anthurium*, it has been reported that *AaMYB2* is highly expressed in the red, pink, and purple spathes of cultivars but barely identifiable in cultivars with white and green spathes (Osorio-Guarín et al., 2021; Liu et al., 2023). *AaMYB3* interacts with *AabHLH1* to regulate proanthocyanidin (PA) accumulation, and several structure genes were co-expressed with *AaMYB3* in red spathes of anthuriums (Li et al., 2019).

Previous studies of anthuriums have been largely focused on specific gene regulations in red colored spathes (Li et al., 2016, Li et al., 2019; Lin et al., 2022), but there have been no reports on key genes in regulation of white and green colorations. Additionally, there has been no information about the interaction between TFs and structure genes in regulation of these two colors. In this study, we hypothesized that the white colored spathe could be due to the increased expression of *LAR* and *ANR*, leading to the production of colorless PA, while the cause of the green spathe could be attributed to the elevated expression of genes related to chlorophyll biosynthesis, and TFs, likely some members of the MYB family could be implicated in the regulation of those key structure genes.

The objectives of this study were to analyze transcript differences among cultivars with different colored spathes through RNA-Seq technology, identify key genes implicated in spathe coloration, test our hypotheses on the occurrence of green and white spathes, and elucidate the relationship between TFs and key structure genes in regulation of the two colors in modern anthurium cultivars.

## Materials and methods

### Plant materials

Eight cultivars of *A. andraeanum* (Hort.) were grown in 20-cm plastic containers filled with a soilless substrate composed of 60% peat mixed with coconut chaff and perlite (STANLEY, Linyi, China) in a shaded greenhouse under a maximum photosynthetic radiation of 200 μmol m^-2^ s^-1^ in Guangzhou Flower Research Center, Guangzhou, Guangdong Province, China. Daytime and nighttime temperatures in the shaded greenhouse were maintained from 25 to 28°C and 19 to 21°C, respectively with a relative humidity ranging from 70 to 90%. Plants were fertilized and watered as described by Chen et al. (2012). Pinching of flower buds was used to promote vegetative growth in the initial flowering stage. Subsequently, plants entered their bloom stage.

Spathes of these cultivars varied in color ranging from red to white. For transcriptomic analysis of spathe colorations, three plants were randomly selected per cultivar, one spathe was collected from each plant at stage 7 of spathe development, i.e., peduncle fully extended but spathe showing full coloration without being open (**Supplementary Figure S1**). Thus, there were three biological replicates per cultivar. To analyze anthocyanin, chlorophyll, and carotenoid levels in fully open spathes, spathes at their developmental stage of 10 (**Supplementary Figure S1**), i.e., spathes were fully expanded but flowers were not dehisced (Collette et al., 2004), were collected (about 200 mg) from three randomly selected plants per cultivar. Collected spathes were immediately extracted with appropriate solutions mentioned below.

### RNA extraction and transcriptomic analysis of spathes

Total RNA was extracted from collected spathes. RNA concentrations were measured using Qubit® RNA Assay Kit in Qubit® 2.0 Flurometer (Life Technologies,CA, USA). RNA integrity was assessed using the RNA Nano 6000 Assay Kit of the Bioanalyzer 2100 system (Agilent Technologies, CA, USA) and also checked by electrophoresis using 1% agarose gel (**Supplemental Table S1**). The mRNA was enriched using magnetic bead with oligo (dT) and used for cDNA synthesis. The first strand cDNA was synthesized using random hexamer primer and M-MuLV reverse transcriptase (Dingguo Changsheng, Inc. Beijing, China). The second strand cDNA synthesis was subsequently performed using DNA polymerase I with RNase H. cDNA fragments were preferentially selected from 150 to 200 bp in length. PCR amplification was performed for 13 cycles as recommended. The library preparation was performed according to the method described by Liu et al. (2021). The cDNA libraries were sequenced by Illumina Novaseq™ system at NextOmics Bio-Tech. Co. (Wuhan, China). Paired-end sequencing of the polyA enriched library was performed on an Illumina Novaseq 6000 platform generating 150 nt reads.

RNA-Seq data were analyzed to identify key structural genes and transcription factors involved in flavonoid biosynthesis and chlorophyll metabolism. Trinity software (version 2.4.0, **Supplementary Table S2**) was used to assemble the short reads (Grabherr et al., 2011). Trinity commands were as follows: Trinity –seqType fq –max_memory 120G –CPU 30 –min_kmer_cov 2 –full_cleanup –left clean.R1.fq.gz –right clean.R2.fq.gz –output trinity.out. Other parameters were default. Based on the sequence similarity principle, TIGR Gene Indices clustering tools (TGICL, version 2.1, **Supplementary File S1**) was further clustered to eliminate redundancy (Pertea et al., 2003). CDS and protein sequences were predicted by Transdecoder software (version 5.7.0, https://github.com/TransDecoder/TransDecoder/releases, default parameters); then, a unigene sequence set of 8 cultivars (24 samples) was obtained (**Supplementary Table S3**). The analysis on the transcriptome assembly to assess completeness was conducted using BUSCO (version 5.4.2) (Manni et al., 2021). The lineage dataset is embryophyta_odb10.

Unigene functions were annotated based on the following database: the NCBI COG/KOG database (http://www.ncbi.nlm.nih.gov/COG/), Kyoto Encyclopedia of Gene and Genomes database (KEGG, https://www.genome.jp/kegg/), non-redundant protein database (NR, ftp://ftp.ncbi.nih.gov/blast/db/), SwissProt protein database (https://www.uniprot.org/), and Gene Ontology (GO, http://geneontology.org/). Identification of flavonoid biosynthesis genes was annotated using KIPEs (Knowledge-based Identification of Pathway Enzymes, https://pbb-tools.de/KIPEs/) based on transcript sequences (Pucker et al., 2020). MYB were also predicted with a dedicated tool MYB annotator (Automatic annotation of MYBs, https://pbb-tools.de/MYB_annotator/) (Pucker, 2022). bHLH were predicted with a dedicated tool bHLH annotator (Automatic annotation of the bHLH gene family in plants, https://pbb-tools.de/bHLH_annotator/) (Thoben and Pucker, 2023). The identifications of flavonoid biosynthesis genes, MYB and bHLH were listed in **Supplementary File S2**.

Gene expression levels were estimated by RSEM software (version 1.1.12) and presented as FPKM (Fragments Per Kilobase of exon model per Million mapped fragments) (Li and Dewey, 2011). Differentially expressed genes (DEGs) were analyzed between different groups by DESeq2 software (version 1.38.3) (Love et al., 2014). DEGs were detected and screened between the eight comparisons, using fold change ≥ 2 and false discovery rate (FDR) ≤ 0.05. The “pheatmap” R package (version 4.2.2, https://www.r-project.org/) was used to determine DEGs abundance, and the scale of heatmap for DEGs were converted from the FPKM was Log2 of the fold change. The enrichment analysis was performed in GO term and KEGG pathway.

For validation of candidate genes associated with the spathe coloration, red spathe cultivars Te Lun Sa (TLS), 2016, and A302; green spathe cultivars Kai Xin Guo (KXG), A166, and A086; and white spathe cultivars Bai Ma (BM), A231BAI, and A168 at stage 7 (**Supplementary Figure S1**) were harvested, immediately immersed in liquid nitrogen, and stored at -80°C for qRT-PCR analysis. These nine cultivars were grown in the same conditions as the aforementioned eight cultivars.

### Extraction and quantification of anthocyanin, chlorophyll, and carotenoid levels

Anthocyanin is red in acid solution. Its color is in proportion to the anthocyanin content. The absorption peak of anthocyanin acid solution is 530, with which molar extinction coefficient is 4.62 × 104. Thus, ultraviolet-visible (UV-Vis) spectrophotometer can be used to measure the anthocyanin content.

To analyze anthocyanin, collected 0.2 g spathes from eight cultivars were extracted in 10 mL of ethanol: hydrochloride (99:1, v/v) at 32°C for 4 h. The sample was centrifuged at 5,000 rpm for 5 min (Centrifuge 5804 R, Eppendorf, Shanghai, China), and the supernatant was stored at 4°C. The residue was extracted with the extraction solution 1 to 2 times until the supernatant turned colorless. Absorbencies were measured at 530 nm and 650 nm wavelengths with an UV-Vis spectrophotometer (UV4800, Unico, Shanghai, China). The relative content of anthocyanin was calculated according to the method (Yang et al., 2022):


$$\text{Anthocyanin }\left(\Delta \text{A}/\text{g}\cdot \text{FW}\right)=\left({\text{A}}_{530}-0.25\times {\text{A}}_{650}\right)/\text{FW}.$$

For quantification of total chlorophyll and carotenoid, fresh spathe samples were ground to powder in a mortar with liquid nitrogen, extracted with 80% acetone, filtered into a test tube, and finally extracted with ethyl acetate. The suspension was centrifuged for 5 min at 2,000 rpm. The absorbance of the supernatant was recorded at 663 nm, 645 nm, and 440 nm, respectively. The total contents of chlorophyll (Chl) and carotenoid were calculated according to the following formula (Zhou et al., 2013; Vaculík et al., 2015):


$${\text{Chl}}_{\text{a}+\text{b}}\left(\Delta \text{A}/\text{g}\cdot \text{FW}\right)={\text{Chl}}_{\text{a}}+{\text{Chl}}_{\text{b}}=20.29\times {\text{A}}_{645}+8.02\times {\text{A}}_{663}.$$

$$\text{Carotenoid }\left(\Delta \text{A}/\text{g}\cdot \text{FW}\right)=0.1\times \left(4.7\times {\text{A}}_{440}-0.27\times {\text{Chl}}_{\text{a}+\text{b}}\right).$$

### Proanthocyanidins extraction and determination

The UV/Vis spectrophotometry was used to evaluate the proanthocyanidins (PA) content. About 200 mg spathe was used for extraction. Soluble PAs from fresh anthurium spathes were extracted and measured by Innovabio Testing Technology Co. (Nanjing, Jiangsu). After ethanol precipitation, the PA content was determined by normal butyl alcohol-hydrochloric acid method at 546 nm. Proanthocyanidins (Cas: 4852-22-6, Yuanye, Shanghai, China) was used as a standard for PA quantification. The content of PA was calculated based on the standard curve according to the method (Li X. et al., 2021).

### Cyanidin and pelargonidin contents measurement

About 100 mg spathe was ground to powder and extracted by ethyl alcohol: distilled water: hydrochloric acid (2:1:1, v/v/v) for supernatant preparation. SCIEX Qtrap6500 mass spectrometer System (AB, Massachusetts, USA) coupled to a LC-30AD high performance liquid chromatograph (Shimadzu, Kyoto, Japan) was used for liquid chromatography-tandem mass spectrometry (LC-MS/MS). Cyanidin chloride (Cas: 528-58-5), and pelargonidin chloride (Cas: 134-04-3, Yuanye, Shanghai, China) was used as a standard for cyanidin and pelargonidin quantification, respectively. The standard substance was dissolved in hydrochloric acid-methanol solution as single standard stock solution. LC-MS/MS analysis was performed by Innovabio Co. (Nanjing, Jiangsu) according to the method of Tomas (2022).

### RNA isolation for qRT-PCR analysis

Spathes at stage 7 (**Supplementary Figure S1**) harvested from the above-mentioned cultivars were ground to a powder with a mortar and a pestle in liquid nitrogen. RNA was extracted using the RaPure Plant RNA Kit (Magen, Guangzhou, China) following the instructions of the manufacturer. Total RNA was quantified using NanodropND1000 UV/Vis spectrophotometer (Thermo Scientific, Walthman, MA), and the purity was assessed by the absorbance ratios of 260/280 nm and 260/230 nm. The integrity of the purified RNA was confirmed with 2% agarose gel electrophoresis. The first-strand cDNA was synthesized from 1 μg of total RNA using a TIANGEN FastKing cDNA Kit (TIANGEN, Beijing, China) according to the manufacturer’s instructions. The 20 μL cDNA samples were diluted to a 1:5 ratio using 80 μL RNase-free water for all gene amplification reactions.

qRT-PCR reactions were performed using SYBR Green detection chemistry by running on 96 well-plates with a CFX connect Real-Time PCR System (BIO-RAD, Shanghai, China) with three replicates for each sample. Primers for each target were designed using Primer3 web service (https://primer3.ut.ee/), and the sequences of all primers are listed in **Supplemental Table S4**. qRT-PCR analysis data were averaged by three biological replicates and calculated using the 2^-△△Ct^ method (Livak and Schmittgen, 2001). All expression levels were normalized with internal control, *AaGAPDH* (Li S. et al., 2021).

### Prediction of MYB binding motifs annotated on promoters

The upstream sequences (5,000 bp) of *AaLAR* (GenBank accession no. OR241534), *AaANR* (GenBank accession no. OR241535), *AaHemB* (GenBank accession no. OR241536), and *AaPor* (GenBank accession no. OR241537) genes were obtained from the *Anthurium* Genome Database of *A. andraenum* cv. Xiaojiao-Texana. Potential binding motifs for TFs of MYB family on *AaLAR*, *AaANR*, *AaHemB*, and *AaPor* promoter regions were predicted by the online website JASPAR^2022^ (http://jaspar.genereg.net/) (Fornes et al., 2019). The TF binding sites and motifs were further screened by a relative profile score threshold of 90%. The binding sites on the positive strand were presented and the common MYB motifs in the anthocyanin or chlorophyll biosynthesis pathway were marked. As the motifs in the JASPAR database were only included in the model species, *Arabidopsis thaliana*, *Oryza sativa*, and *Zea mays*, the MYB proteins in *A. andraeanum* were aligned together by Clustalw for conducting phylogenetic analysis using the neighbor-joining method in MEGA version 7.0 to determine the genetic relationship (**Supplementary Figures S2**, **S3**).

### Statistical analysis

Data for pigment contents and gene relative expression levels were statistically analyzed using SPSS (SPSS statistics 19, IBM Corp., Armonk, NY, USA). If significance occurred among cultivars, means were separated using Fisher’s LSD at *P<* 0.05 level. Data are presented as mean ± S.E. with either 3 or 5 replicates.

## Results

### Spathe pigment contents of eight cultivars

**Figure 1:**
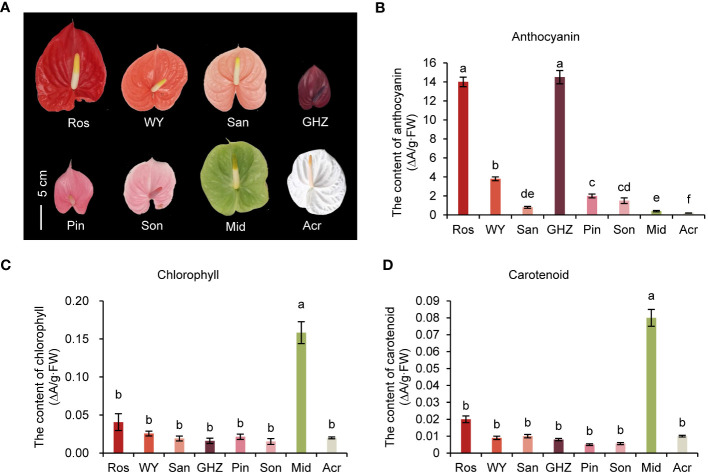
Spathe phenotype and pigment analysis of eight anthurium cultivars. Phenotypes of anthurium spathes **(A)**, the total content of anthocyanin **(B)**, chlorophyll **(C)**, and carotenoid **(D)** in spathes of eight cultivars are shown as means with corresponding standard error (n = 5). Different letters above bars indicate significant differences analyzed by Fisher’s LSD test at *P<* 0.05 level.

Spathes of eight anthurium cultivars had eight different colors, including ‘Rosa’ (Ros) with red spathe; ‘Wu Yang’ (WY), orange spathe; ‘Sante’ (San), coral spathe; ‘Guang Hua Zi Yun’ (GHZ), purple spathe; ‘Pink Champion’ (Pin), pink spathe; ‘Sonate’ (Son), light pink; ‘Midori’ (Mid), green spathe; and ‘Acropolis’ (Acr) (**Figure 1A**). Anthocyanin contents in spathes of Ros and GHZ were the highest, 14.0 and 15.0 ΔA/g·FW, respectively, which were substantially greater than the others (**Figure 1B**). Anthocyanin content in Acr was the lowest, only 0.5 ΔA/g·FW. The other cultivars had anthocyanin ranging from 1.0 to 3.8 ΔA/g·FW, suggesting that anthocyanin contents were associated with redness of spathes. The main component of anthocyanins in GHZ was cyanidin, up to 0.25 mg·g^-1^·FW, rather than pelargonidin, which was only 0.025 mg·g^-1^·FW (**Supplementary Figure S4A**). Proanthocyanidin (PA) contents of Ros and San were highest, 11.4 and 14.5 mg·g^-1^·FW, respectively, following by Son and Mid. PA content in Acr was about 5.27 mg·g^-1^·FW (**Supplementary Figure S4B**).

The Chl and carotenoid levels were the highest in Mid at 0.16 ΔA/g·FW and 0.08 ΔA/g·FW, respectively (**Figures 1C, D**). Notably, the contents of both Chl and carotenoid in the other seven cultivars were not significantly different, ranging from 0.02 to 0.04 ΔA/g·FW for Chl, and 0.002 to 0.02 ΔA/g·FW for carotenoid, respectively. These results showed that the pigment composition and content in green and white spathes were different from those of the other colored spathes.

### Transcriptome sequencing and annotation

Raw data or reads generated from the transcriptome sequencing of 24 samples (8 cultivars, each with three biological replicates) ranged from 40,964,478 to 63,961,814. After stringent quality control checks and filtering, a total of 1,947,453,054 paired-end clean reads were obtained with Q30 values higher than 92% in each sample library. The data volume per library was greater than 6 Gb. The clean reads were spliced and assembled using Trinity. N50 length of the Trinity assembly ranged from 1610 to 1799 bp (**Supplementary Table S2**). Trinity sequence of all samples were merged. According to the sequence similarity principle, the merged Trinity assembly sequences were clustered for a set of unigene sequences, which resulted in a total of 62,013 unigenes. The length of assembled unigenes varied from 220 to 21,222 bp. The average length of the unigenes was 2,038.45 bp, and the N50 was 2,519 bp (**Supplementary Figure S5A**, **Supplementary Table S3**). These data showed that the throughput and sequencing quality were high enough for further analysis. The complete BUSCOs of the transcriptome assembly was 1387, accounting for 85.9% (**Supplementary Figure S6**). In this study, the tissue used for RNA-Seq was spathes only. Some genes were hardly detectable in the spathe, suggesting that some genes were not expressed in this special organ. Thus, it was reasonable for the incompleteness of this transcriptome assembly.

A total of 48,582 coding sequences (CDS) were predicted, and their lengths varied from 261 to 15,300 bp with the majority ranging from 972 to 1,206 bp (**Supplementary Figure S5B**). These results implied that the reads derived from transcriptome sequencing had high quality and reliability. Further, 33,392 unigenes were assigned to 23 categories in the KOG database (**Supplementary Figure S5C**). Among them, 6,806 unigenes, “Posttranslational modification, protein turnover, chaperones” represented the largest group with 3,453 unigenes, followed by “Signal transduction mechanisms” (2,858 unigenes)”, and Transcription (1,965 unigenes). In the NR database, the identified unigenes were used to detect their homologous genes in other species by blast, and the results showed that the ratio of homologous genes in *Elaeis guineensis*, *Phoenix dactylifera*, *Nelumbo nucifera*, *Musa acuminata* subsp. *Malaccensis*, and *Ananas comosus* were 25.60%, 21.40%, 8.53%, 5.92%, and 4.59%, respectively (**Supplementary Figure S5D**).

### Differentially expressed genes among different colored spathes

Transcriptome analysis identified differentially expressed genes (DEGs) in different colored spathes based on their FPKM (Fragments Per Kilobase of exon model per Million mapped fragments) levels. To identify the key DEGs involved in different colorations, seven pair-wise comparisons were conducted which included Ros vs. Acr; WY vs. Acr; San vs. Acr; Mid vs. Acr; GHZ vs. Acr; Pin vs. Acr; and Son vs. Acr (**Supplementary Figure S7A**). Among these comparisons, the greatest abundance of DEGs (29,588) was found in Pin and Acr libraries, of which 13,891 and 15,769 genes were up-regulated and down-regulated, respectively. Conversely, the lowest abundance of DEGs (14,015) were recorded in GHZ and Acr libraries (**Supplementary Figure S7A**). Gene functional annotation was carried out to understand the function of DEGs. According to the GO annotation, a total of 51,469 DEGs were spread across 1,123 terms consisting of three domains: molecular functions accounting for 57.52%, cellular components 12.54%, and biological processes 29.94%. The most frequent terms under the molecular function were protein binding, ATP binding, protein kinase activity, and nucleic acid binding, DNA binding, and zinc ion binding (**Supplementary Figure S7B**). To further examine the metabolic process of the DEGs, the KEGG pathway for DEGs in seven comparison pairs were conducted. The top 20 enriched pathways were listed in all comparable groups (**Supplementary Figure S7C**). From the results of KEGG enrichment analysis, the pathways for flavonoid biosynthesis, and porphyrin and chlorophyll metabolism were enriched in the comparisons of Ros vs. Acr; GHZ vs. Acr; WY vs. Acr; and Mid vs. Acr (**Supplementary Figures S7C**, **S8**).

### Differentially expressed genes in the flavonoid biosynthesis pathway

**Figure 2:**
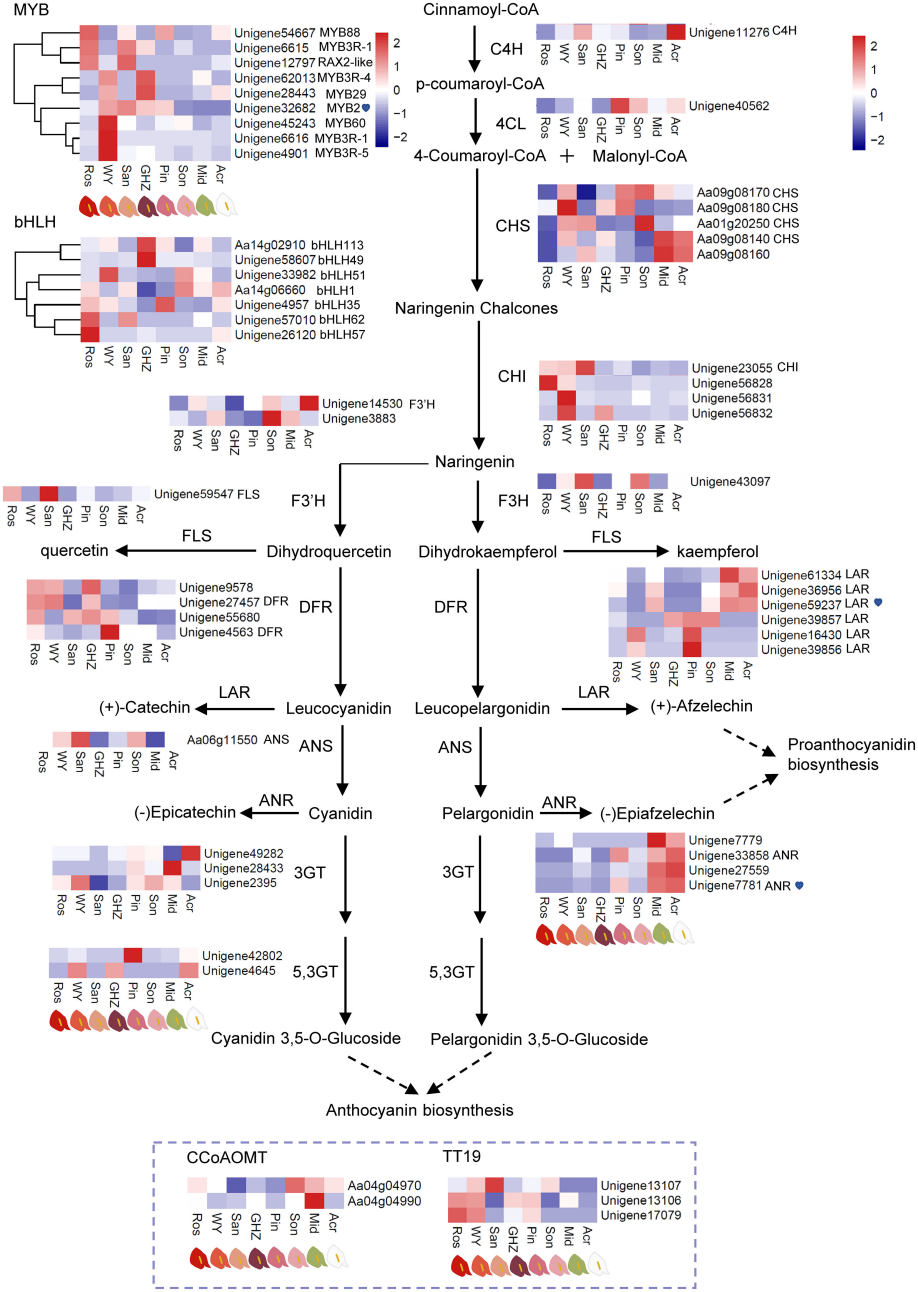
DEGs involved in the flavonoid metabolism and their expression levels. Heatmaps were constructed based on Fragments per kilobase per million mapped reads (FPKM) of eight anthurium cultivars with different spathe colors. Color bar: Log2 (fold changes).

The DEGs associated with the flavonoid biosynthesis were analyzed through the examination of KEGG enrichment and gene annotations, from which 56 DEGs in the flavonoid pathway were identified, including nine *MYB* genes and seven *bHLH* genes regulating flavonoid biosynthesis, and one *C4H*, one 4-coumarate-CoA ligase-like (*4CL*), five *CHS*, four *CHI*, one *F3H*, two *F3’H*, one flavonol synthase (*FLS*), four *DFR*, six *LAR*, one *ANS*, three anthocyanidin 3-O-glucosyltransferase (*3GT*), two anthocyanidin 5,3-O-glucosyltransferase (*5,3GT*), four *ANR*, two caffeoyl-CoA O-methyltransferase (CCoAOMT), and three glutathione S-transferase F12 (also named TT19) in seven unrepetitive comparison pairs of Ros, WY, San, GHZ, Pin, Son, Mid, and Acr. According to the expression level of DEGs shown in heatmap (**Figure 2**), *MYBs* were highly expressed in Ros, WY, San, and GHZ compared to their expression in the other cultivars, of which *MYB2 Unigene32682* exhibited notable changes with the cultivars or the content of anthocyanin in spathes (**Figure 2**, **Supplementary Figure S9**). The results implied that *MYB* genes could play an important role in regulation of anthocyanin biosynthesis in anthuriums. For *CHI*, its expression levels in Pin, Son, Mid, and Acr were relatively lower than in other cultivars (**Figure 2**), indicating that more naringenin chalcones was transformed into naringenin in Ros, WY, San, and GHZ. The expression level of *F3’H* in Son was significantly higher than other spathes, suggesting that more dihydroquercetin (DHQ) was synthesized in Son than the other spathes. *F3H* gene expression in San was the highest among eight anthurium cultivars, indicating that naringenin in San was catalyzed into more dihydrokaempferol (DHK). Higher levels of *DFR* expression occurred in Ros, WY, and GHZ, converting more dihydroquercetin/dihydrokaempferol to leuco-cyanidin/pelargonidin. The expression of *ANS* was increased in WY, San, and Son, with cyanidin and pelargonidin accumulation. The gene expression of *5,3GT* was highly detected in WY and GHZ, accompanied with 3,5-O-glucoside product. Downregulation of a decorating enzyme *CCoAOMT* gene seemed to activate the anthocyanin biosynthesis in red- and purple-colored spathes. Anthocyanin transport carrier *TT19* was induced in Ros, WY, San, GHZ, Pin, and Son, supporting anthocyanin accumulation and especially cyanidin accumulation. The increased expression of the *LAR* gene, such as *Unigene59237* (**Figure 2**), could result in converting leucocyanidin and leucopelargonidin to catechin and afzelechin, respectively. The highly expressed *ANR* gene, especially *Unigene7781*, could make cyanine and pelargonidin flow into epicatechin and epiafzelechin, respectively. The results showed that the expression of *LAR* and *ANR* were significantly different in green and white spathe cultivars compared with the six other cultivars. The increased expression of *LAR* and *ANR* could reduce the catalytic substrate to synthesize anthocyanins, resulting in accumulation of proanthocyanidins in spathes.

### Transcript factors and key structural genes related to flavonoid biosynthesis

**Figure 3:**
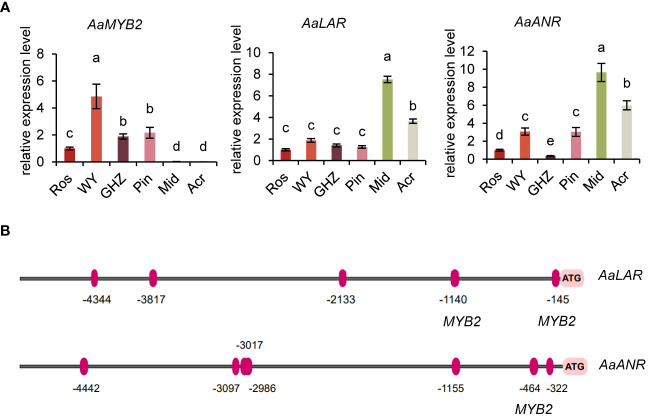
Molecular analysis of key genes in the flavonoid biosynthesis pathway. **(A)** Expression analysis of *AaMYB2*, *AaLAR*, and *AaANR* in the spathe tissue of six cultivars. Expression levels were normalized based on the expression of the *GADPH* gene. Data are presented as means of three biological replicates and error bar shows standard error (n = 5). Different letters above bars indicate significant differences determined by Fisher’s LSD test at *P<* 0.05 level. **(B)** Prediction of MYB2 transcription factor binding sites in the promoters of *AaLAR* and *AaANR*. The binding sites were shown as red ovals (positive strand).

Except for differential expression of flavonoid biosynthesis genes, we also analyzed if MYB-TFs were implicated in the regulation of some of the genes. A *MYB*-TF (*Unigene32682*, *MYB2*), a *LAR* gene (*Unigene59237*), and a *ANR* gene (*Unigene7781*) were chosen from DEGs to analyze their expressions in the eight cultivars (**Figure 2**). The transcript level of *MYB2* was significantly lower in the green or white spathed cultivar compared with the color intensified ones. Conversely, in comparison to the color intensified spathes, green and white spathes had significantly high levels of *LAR* and *ANR* transcripts (**Figure 3A**, **Supplementary Figure S10**).

Promoter analysis of *LAR* and *ANR* genes showed that the promoter of *LAR* contained two motifs bound by *MYB2*, and the promoter of *ANR* also had one motif bound by *MYB2* (**Figure 3B**, **Supplementary Figure S2**), implying that the expression levels of *LAR* and *ANR* could be likely regulated by the TF-*MYB2* (**Figure 3B**). In addition, expression analysis revealed that the expression pattern of *MYB2* was opposite to that of *LAR* and *ANR*, suggesting *MYB2* might negatively regulate the expression of *LAR* and *ANR* (**Figure 3A**).

*bHLH* family members were also analyzed. Expression levels of *bHLH113* and *bHLH49* increased in purple spathe GHZ, while *bHLH62* and *bHLH57* were highly detected in red spathe Ros (**Figure 2**), indicating bHLH might be involved in the positive regulation of anthocyanin biosynthesis.

### Transcription factors and key structural genes related to chlorophyll biosynthesis

**Figure 4:**
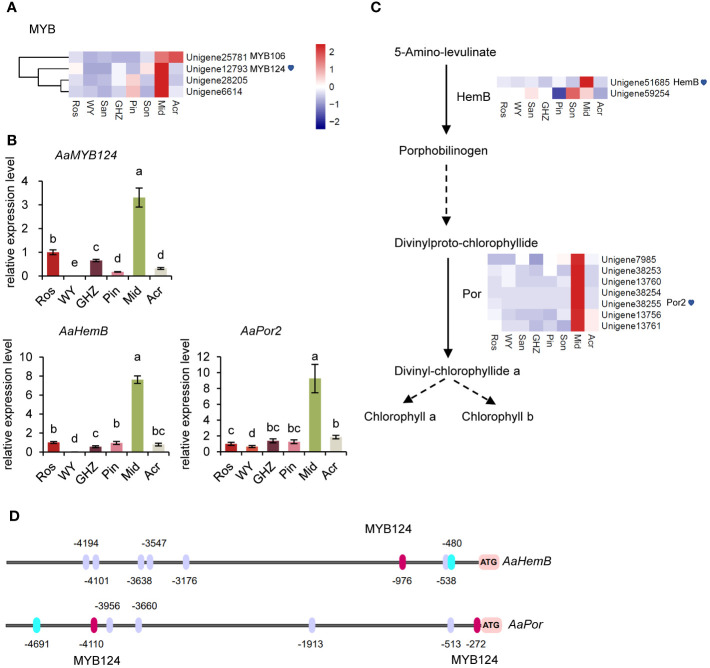
Molecular analysis of key genes in the chlorophyll metabolism pathway. **(A)** Heatmap of *MYBs* related to chlorophyll metabolism. **(B)** Expression analysis of *AaMYB124*, *AaHemB*, and *AaPor* in the spathe tissue of six cultivars. Expression levels were normalized to the expression of the *GADPH* gene. Data are presented as means of three biological replicates and error bar shows standard error (n = 5). Different letters above bars indicate significant differences analyzed by Fisher’s LSD at *P<* 0.05 level. **(C)** DEGs involved in chlorophyll metabolism and their expression levels. Each colored cell represents the average FPKM value standardized by the Z-Score of each gene. **(D)** Prediction of *MYB124* transcription factors binding sites in the promoters of *AaHemB* and *AaPor*. The binding sites were shown in red (*MYB124*) and purple ovals (positive strand).

Comparative analysis of DEGs showed that the expression levels of a set of MYB-TFs, including *MYB124* (*Unigene12793*) were significantly higher in Mid (green spathe) than in the others, which could suggest that those MYB-TFs may also participate in the regulation of Chl synthesis (**Figure 4A**). Thus, the DEGs in the chlorophyll biosynthesis pathway were further analyzed, and data indicated that the expressions levels of *HemB* (*Unigene51685*) and *Por* (*Unigene38255*) were significantly greater in Mid than in the other cultivars (**Figure 4B**, **Supplementary Figure S10**). To explore the expression pattern of the DEGs, qRT-PCR was conducted with three genes, *MYB124*, *HemB*, and *Por*, involved in the biosynthesis of Chl. The results showed that the relative expression levels of the three genes were similar to the FPKM values (**Figure 4C**), suggesting that *MYB124* was positively correlated with the expression of *HemB* and *Por* in green spathe. These data implied that *MYB124* could promote Chl synthesis by regulating *HemB* and *Por* genes.

To further investigate the regulatory relationships among *MYB124*, *HemB*, and *Por*, we analyzed the promoter sequences of *HemB* and *Por*, respectively. The analysis showed that upstream of *HemB* and *Por* contained MYB124-bounding motifs, respectively (**Figure 4D**, **Supplementary Figure S3**). The results supported the above analysis that TF-*MYB124* enhanced the expression of the downstream genes, *HemB* and *Por* and promoted Chl accumulations, thus the green spathe in cultivar Mid.

### Differentially expressed genes in the carotenoid biosynthesis pathway

Since Mid with green spathe was loaded with carotenoids (**Figure 1D**), we took a closer look at gene expression in carotenoid biosynthesis pathway. Five unigenes were identified, including one *PDS* (Unigene54090), two *LUT5* (Unigene14810 and Unigene58790), one *CrtR-b* (Unigene54067), and one *ZEP* (Unigene8259). Upstream gene phytoene desaturase (PDS), which is associated with carotenoid content, was detected to be highly expressed in Mid. Increased expression of lutein deficient (*LUT5*) and β -carotene hydroxylase (*CrtR-b*) in Mid suggested more lutein accumulation (**Supplementary Figure S11**). Meanwhile, the level of zeaxanthin epoxidase (*ZEP*) was significantly greater in Mid than other cultivars, indicating that *ZEP* may be involved in carotenoid synthesis through the epoxidation of β-carotene and β-cryptoxanthin (**Supplementary Figure S11**). Thus, increased carotenoid content in Mid might be caused by these elevated expression genes in carotenoid biosynthesis pathway.

### qRT-PCR analysis of candidate genes

**Figure 5:**
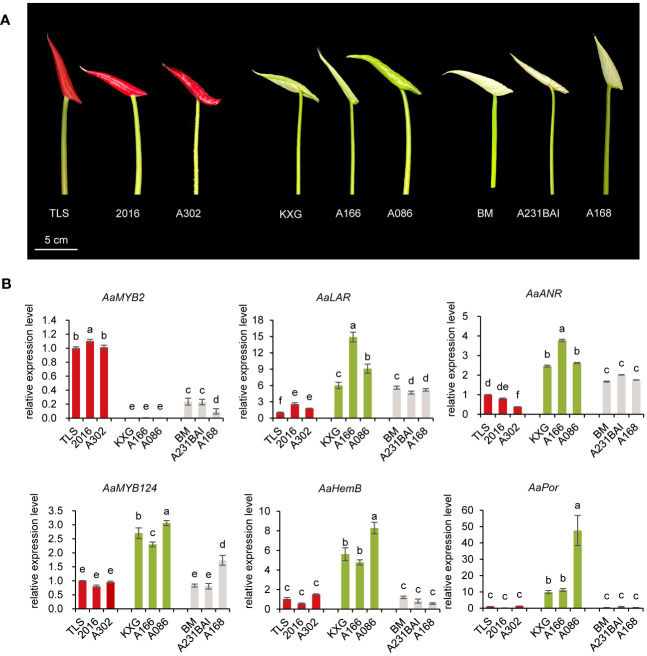
Expression analysis of candidate genes in nine additional anthurium cultivars with different spathe colors. **(A)** Spathe phenotypes of red cultivars TLS, 2016, A302, green cultivars KXG, A166, A086, and white cultivars BM, A231BAI, and A168 at early bud (S7 stage). **(B)** The transcript level of *AaMYB2*, *AaMYB124*, *AaLAR*, *AaANR*, *AaHemB*, and *AaPor* in the spathe tissue of nine cultivars. Expression levels were normalized based on the expression of the *GADPH* gene. Data are presented as means of three biological replicates and the error bar shows standard error (n = 5). Different letters above bars indicate significant differences analyzed by Fisher’s LSD test at *P<* 0.05 level.

To further confirm key genes involved in the change of spathe colors, we used qRT-PCR to analyze the expression pattern of seven candidate genes in nine additional cultivars with red, green, and white spathes at S7 stage (**Figure 5**, **Supplementary Figure S1**). As expected, the expression levels of *LAR* and *ANR* in green or white spathes were significantly higher than in red spathes. In contrast, *MYB2* gene exhibited the opposite expression pattern of *LAR* and *ANR* (**Figure 5**, **Supplementary Figure S9**). Therefore, these results confirmed that the expression of *MYB2* was negatively correlated with the expression of *LAR* and *ANR*.

*HemB* and *Por* genes were highly expressed in green spathes but at very low levels in red and white spathes (**Figure 5**). Such expression levels were closely associated with Chl contents in the respective spathes (**Figure 1C**). Additionally, *MYB124* was highly correlated with *HemB* or *Por* expression and the total Chl content, suggesting that *HemB* and *Por* may be regulated by *MYB124* in regulation of Chl biosynthesis.

Taken together, these results showed that the white colored spathe was probably due to the increased expression of *LAR* and *ANR*, resulting in the production of colorless PA. The green-colored spathe was likely attributed to the elevated expression of *HemB* and *Por*, leading to chlorophyll biosynthesis. While *MYB2* could negatively regulate the expression of *LAR* and *ANR*, and *MYB124* enhanced the expression of *HemB* and *Por*.

## Discussion

Modifying spathe colors has been a major emphasis in anthurium breeding since the 1950s (Kamemoto and Nakaone, 1955; Kamemoto and Kuehnle, 1996). Later, Elibox and Umaharan (2008) reported that three dominant genes, *O*, *R*, and *M* control spathe colors. Plants with *O*_*R*_ genotypes produce colorful spathes, while those with *ooR*_ or *O_rr* give rise to white spathes. Elibox and Umaharan (2008) proposed that *R* and *O* encoded transcription factors regulating *CHS*, *F3H*, *AN*S, and *DFR* expression, respectively, and *M* encoded *F3’H* (flavonoid 3’-hydroxylase) that catalyzes dihydrokaempferol to dihydroquercetin. Subsequent analyses of selected genes at mRNA and protein levels indicated that *F3H* and *ANS* were probably controlled by a transcription factor encoded by *R* locus, and *CHS* could be controlled by a different mechanism (Gopaulchan et al., 2014). These studies provide classical genetic and molecular information on anthocyanin biosynthesis in modern anthuriums. With the advent of RNA-Seq technology, Li et al. (2016) isolated an *R2R3-MYB* gene, *MYB2* in *A. andraeanum* (Hort.) and found that *MYB2* plays an important role in spathe accumulation of anthocyanin by positively regulating *CHS*, *ANS*, and *F3’H* expressions. Furthermore, Li et al. (2019) transformed *AaMYB3* from *A. andraeanum* (Hort.) into tobacco and found that *AaMYB3* upregulated *NtDFR* and *NtANS*. Although the present study was primarily focused on white and green spathes, our analysis of eight cultivars with different colors showed that cultivars with red colored spathes (Ros, WY, and GHZ) generally had higher contents of anthocyanin. The expression of *MYB2* gene in Ros, WY, and GHZ was mostly higher than other cultivars (**Figures 1**, **2**). Our results agree with the report of Li et al. (2016) that *MYB* genes are critical for anthocyanin accumulation in reddish spathes of anthuriums. Phylogenetic analysis suggests that *AaMYB2* was highly similar to *AtMYB113*. *AtMYB113* of *Arabidopsis* has been mentioned in activating anthocyanin synthesis (Gonzalez et al., 2008). Thus, *MYB2* gene was selected as a target gene as the subsequent analysis. The present study explored the molecular basis of white colored spathes. In the flavonoid pathway, anthocyanidins can be catalyzed by ANR to produce epicatechin, and leucoanthocyanidins are catalyzed by LAR to synthesize catechin (Akagi et al., 2009), which are important steps in the biosynthesis of colorless proanthocyanidins (Bogs et al., 2007; Nie and Stürzenbaum, 2019). Our data showed that anthocyanin was barely detectable, and chlorophyll and carotenoid contents were lower in white spathe (Acr). Correspondingly, the expression of *AaMYB2* in white spathes was extremely lower, but the expression levels of *AaLAR* and *AaANR* were higher in white spathes (**Figures 2**, **3**). These data suggest that the decreased expression of *AaMYB2* is correlated with the activation of *LAR* and *ANR* expressions. Increasing expression of *LAR* and *ANR* genes could enhance proanthocyanin production, and in turn alter the competition between anthocyanin and proanthocyanidin biosynthesis, resulting in white colored spathes. Similar results were reported in other crops as well. For example, transcript levels of *LAR* and *ANR* genes were higher in white than red fruits in strawberry (Salvatierra et al., 2013). The transcript accumulation of *MmANR* gene leads to the abundant catechin and epicatechin in white flowers in *Michelia maudiae* (Lang et al., 2019). Overexpression of *ANR* gene promotes the proanthocyanidins biosynthesis in tobacco flowers (Han et al., 2012). These reports concur with our results that increasing expression of *LAR* and *ANR* genes is associated with organ discoloration.

We also investigated molecular underpinnings of green colored spathes. Similar to the white spathe, anthocyanidins were hardly detected in green spathes (**Figure 1**). This could be attributed to the lower expression of *MYB2* and increased expression of *LAR* and *ANR* (**Figure 3**), resulting in an extremely low anthocyanin content. In addition, our data showed that the expression level of *AaMYB124* was higher, which was associated with the increased expression of *HemB* and *Por* and elevated contents of chlorophyll (**Figures 4**, **5**). These results suggest that *AaMYB124* may promote chlorophyll synthesis by positively regulating *HemB* and *Por* expression in green spathe. It was worth to pay more attention to the transcription factor *AaMYB124* in enhancing the transcription of chlorophyll metabolism genes thus green spathe. Therefore, the formation of green spathes is not only dependent on the accumulation of a large amount of chlorophyll via upregulating *HemB* and *Por* by *AaMYB124*, but also related to the high expression level of *AaLAR* and *AaANR*, which limits anthocyanin biosynthesis.

**Figure 6:**
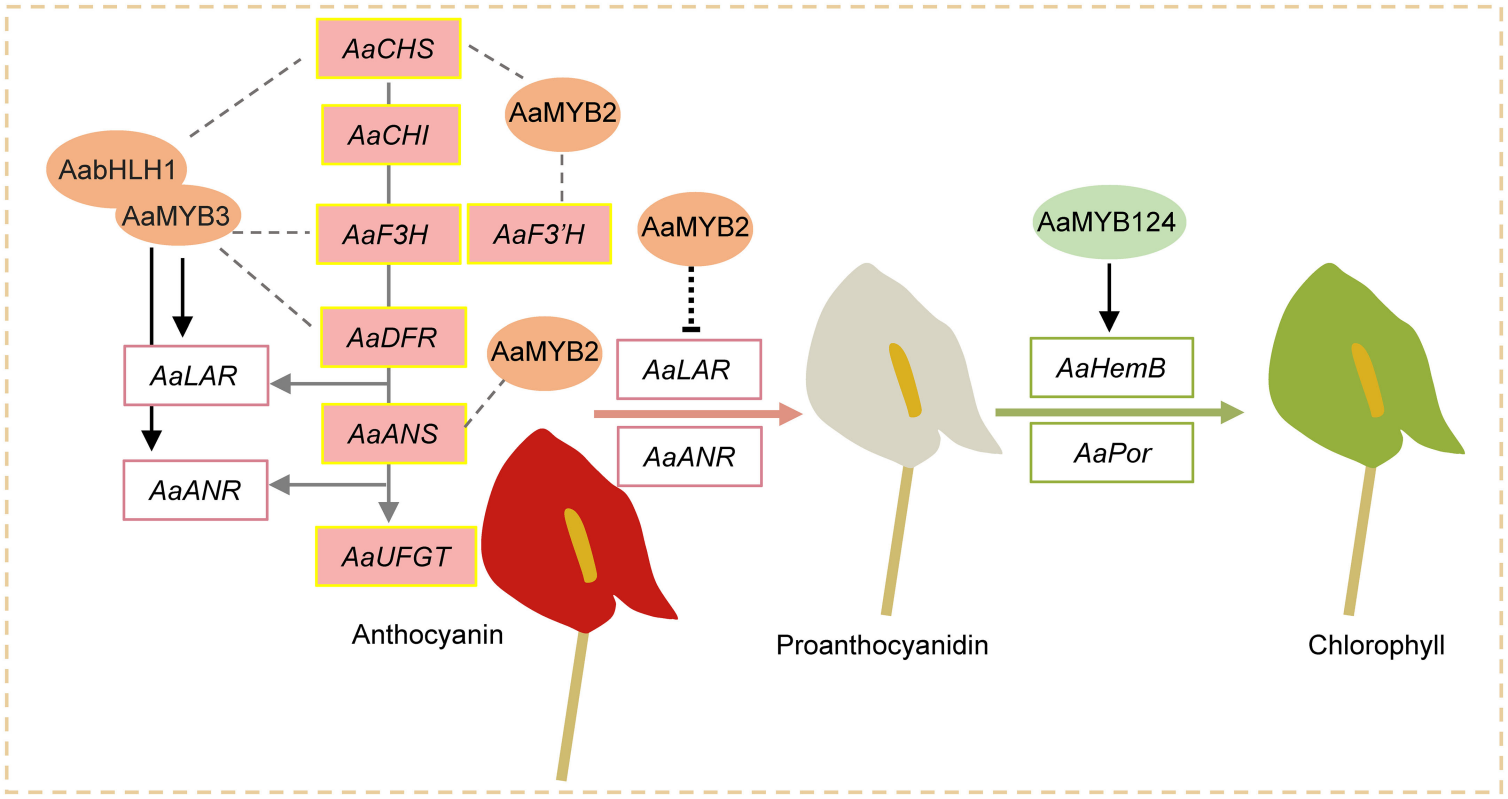
A working model for the formation of red, white, and green spathes in *A. andraeanum* (Hort.). AaMYB3 interacts with AabHLH1 to regulate proanthocyanidin accumulation. *AaMYB3* was coexpressed with *AaCHS*, *AaF3H*, *AaDFR*, *AaANS*, *AaLAR*, and *AaANR* in the developing red spathe. The different expression of *AaUFGT* might explain the formation of anthocyanin-loss mutant. Increased expression of *AaMYB2* enhances the expression of *AaCHS*, *AaF3’H*, and *AaANS* as well as anthocyanin accumulation in reddish spathes. Meanwhile, decreased expression of *AaMYB2* activates *AaLAR* and *AaANR* expression and promotes biosynthesis of proanthocyanidins, leading to spathes with white color. Under the suppressed expression of *AaMYB2*, two other TFs *AaMYB124* upregulated *HemB* and *Por* expression, thus chlorophyll accumulation and spathe with green coloration.

Considering the above results, we proposed a working model to illustrate the formation of red, white, and green spathes in *A. andraeanum* (Hort.) (**Figure 6**). *AaMYBs* play important roles in regulation of pigment biosynthesis. Increased expression of *AaMYB2* enhances the expression of *AaCHS*, *AaF3’H*, and *AaANS* and anthocyanin biosynthesis, resulting in the formation of reddish spathes. On the other hand, the decreased expression of *AaMYB2* is associated with the increased expression of *AaLAR* and *AaANR*, which could block anthocyanin synthesis and promote biosynthesis of proanthocyanidins, leading to spathes with white color. Under the decreased expression of *AaMYB2*, the formation of green colored spathes is attributed to the increased expression of *AaMYB124* as it promotes the expression of both *HemB* and *Por*, thus chlorophyll biosynthesis and the greenish spathes. This working model slightly differs from the report of Gopaulchan et al. (2014) that *F3H* and *ANS* were controlled by a TF encoded by *R* locus, but *CHS* could be controlled by a different mechanism. However, this model concurs with the report of Li et al. (2016) that *AaMYB2* enhances the accumulation of anthocyanin in spathes by positively regulating *CHS*, *ANS*, and *F3’H*. Li et al. (2019) also mentioned that *AaMYB3* was associated with *NtLAR* and *NtANR* in tobacco, resulting in a lighter color by reducing anthocyanin level and enhancing proanthocyanidin accumulation. As far as is known, this is the first attempt to elucidate the likely mechanisms underlying the formation of white and green colored spathes in modern anthuriums, i.e., their formations are regulated by *AaMYBs*: the decreased expression of *AaMYB2* and the increased expressions of *AaMYB124.* Further research will be conducted to test this model.

Our effort on elucidating the molecular basis behind the spathe coloration is intended to improve modern anthuriums through genetic manipulation. There were about 14 reported anthurium transformations in the literature, but none of transformed genes are TFs (Teixeira da Silva et al., 2015). On the other hand, understanding the role of TFs and genetic transformation of the TFs could substantially alter plant phenotypes. For example, transformation of a member of R2R3-MYB gene *VvMYBA1* from grape plants into grape, tobacco, and *Ficus lyrata* produced purple-leaved grape and purple flowered tobacco (Li et al., 2011) as well purple and variegated leaved fiddle fig plants (Zhao et al., 2013). With the availability of anthurium transformation systems and a better understanding of TFs involved in the pigment biosynthesis, genetic transformation or gene editing of TFs could be a viable approach for modification of spathe colors in modern anthuriums.

## Conclusions

This study tested anthocyanin, chlorophyll, and carotenoid contents in different colored spathes of eight cultivars and analyzed their transcript differences using RNA-Seq. Anthocyanin was generally higher in red spathes depending on the cultivar, the elevated level of chlorophyll occurred only in green spathes, but both anthocyanin and chlorophyll were extremely lower in white spathes. Thus, RNA-Seq analysis was focused on flavonoid biosynthesis and chlorophyll metabolism pathways. Results showed that the decreased expression of *AaMYB2* was associated with increased expression of *AaLAR* and *AaANR*, which led to the conversion of anthocyanidins to colorless proanthocyanidin, thus white spathes. Meanwhile, the decreased expression of *AaMYB2* was accompanied with increased expression of *AaMYB124*, and their expression upregulated *AaHemB* and *AaPor*, key genes in chlorophyll metabolism, thus green spathes. A working model for the formation of red, white, and green spathes in modern anthuriums was proposed, which will be verified in further studies.

## Data availability statement

The data presented in the study are deposited in the NCBI repository, accession number of Ros (SAMN34164551), WY (SAMN34164548), San (SAMN34164547), GHZ (SAMN34164553), Pin (SAMN34164550), Son (SAMN34164549), Mid (SAMN34164552), and Acr (SAMN35719425) available at: https://submit.ncbi.nlm.nih.gov/about/sra/, and AaLAR (OR241534), AaANR (OR241535), AaHemB (OR241536), and AaPor (OR241537) available at: https://submit.ncbi.nlm.nih.gov/about/bankit/.

## Author contributions

Writing-original draft, JL and QT; formal analysis, JL and LZ; funding acquisition, MY; methodology, ZY; data curation, ZY; resources, QX; investigation, LZ; project administration, XZ; interpretation, writing-review and editing, X-QZ and JC; supervision, HG. All authors contributed to the article and approved the submitted version.
