# Supplementary material for: Identification of key genes responsible for green and white colored spathes in Anthurium andraeanum (Hort.)
Source: Front Plant Sci. 2023 Sep 6;14:1208226. doi: 10.3389/fpls.2023.1208226 (PMC10511891; doi:10.3389/fpls.2023.1208226)
Supplement: Supplementary file 1 [file DataSheet_1.docx]

Supplementary Material

Identification of key genes responsible for green and white colored spathes in *Anthurium andraeanum* (Hort.)

Jieni Li^1+^, Quanya Tan^2+^, Maosheng Yi^3^, Zhengnan Yu^1^, Qing Xia^3^, Lu Zheng^1^, Jianjun Chen^5^, Xiaoyun Zhou^3 *^, Xiang-Qian Zhang^1, 4*^, Herong Guo^1*^

^1^College of Forestry and Landscape Architecture, South China Agricultural University, Guangzhou 510642, China

^2^Guangdong Provincial Key Laboratory of Plant Molecular Breeding, South China Agricultural University, Guangzhou 510642, China

^3^Guangzhou Flower Research Center, Guangzhou 510300, China

^4^School of Food Science and Engineering, Foshan university, Foshan 528000, China

^5^Mid-Florida Research and Education Center, Environmental Horticulture Department, Institute of Food and Agricultural Sciences, University of Florida, Apopka, Florida 32703, USA

+ These authors contributed equally to this work.

*** Correspondence:**

xyzh28@126.com (Xiaoyun Zhou)

aacrav@163.com (Xiang-Qian Zhang)

guohe-rong@scau.edu.cn (Herong Guo)

**TABLE S1** RIN data of total RNA used for RNA-Seq analysis of eight Anthurium cultivars.

| Sample | RIN | Sample | RIN |
| --- | --- | --- | --- |
| Ros- RNAs1 | 8.8 | Pin- RNAs1 | 8.5 |
| Ros- RNAs2 | 8.6 | Pin- RNAs2 | 8.9 |
| Ros- RNAs3 | 8.7 | Pin- RNAs3 | 8.8 |
| WY- RNAs1 | 8.9 | Son- RNAs1 | 8.5 |
| WY- RNAs2 | 8.9 | Son- RNAs2 | 8.3 |
| WY- RNAs3 | 8.8 | Son- RNAs3 | 8.3 |
| San-RNAs1 | 8.6 | Mid - RNAs1 | 8.5 |
| San-RNAs2 | 8.5 | Mid - RNAs2 | 8.7 |
| San-RNAs3 | 8.6 | Mid - RNAs3 | 8.8 |
| GHZ -RNAs1 | 8.7 | Acr- RNAs1 | 7.7 |
| GHZ -RNAs2 | 8.7 | Acr- RNAs2 | 8.5 |
| GHZ -RNAs3 | 8.8 | Acr- RNAs3 | 8.3 |

Note: Ros, WY, San, GHZ, Pin, Son, Mid, and Acr are abbreviations of Anthurium cultivars Rosa, Wu Yang, Sante, Guang Hua Zi Yun, Pink Champion, Sonate, Midori, and Acropolis, respectively. RNAs 1, 2, and 3 indicate three biological replicates.

**TABLE S2** The result of Trinity assembly.

| Sample | Total Length | Total number | Average Length | N50 Length |
| --- | --- | --- | --- | --- |
| Ros_RNAs_1 | 130578821 | 130712 | 998.98 | 1715 |
| Ros_RNAs_2 | 121756519 | 123936 | 982.41 | 1680 |
| Ros_RNAs_3 | 158460965 | 167825 | 944.2 | 1670 |
| WY_RNAs_1 | 86216746 | 88937 | 969.41 | 1636 |
| WY_RNAs_2 | 122796711 | 129682 | 946.91 | 1668 |
| WY_RNAs_3 | 115350826 | 121708 | 947.77 | 1676 |
| San_RNAs_1 | 106310153 | 109314 | 972.52 | 1682 |
| San_RNAs_2 | 135643618 | 151072 | 897.87 | 1610 |
| San_RNAs_3 | 108129931 | 105533 | 1024.61 | 1761 |
| GHZ_RNAs_1 | 131624638 | 137132 | 959.84 | 1676 |
| GHZ_RNAs_2 | 153457643 | 166916 | 919.37 | 1640 |
| GHZ_RNAs_3 | 140163722 | 148663 | 942.83 | 1663 |
| Pin_RNAs_1 | 138746992 | 141684 | 979.27 | 1717 |
| Pin_RNAs_2 | 131115977 | 135211 | 969.71 | 1693 |
| Pin_RNAs_3 | 119963786 | 123986 | 967.56 | 1678 |
| Son_RNAs_1 | 92968851 | 94513 | 983.66 | 1643 |
| Son_RNAs_2 | 92383023 | 91480 | 1009.87 | 1680 |
| Son_RNAs_3 | 109875820 | 109709 | 1001.52 | 1731 |
| Mid_RNAs_1 | 137591433 | 137658 | 999.52 | 1766 |
| Mid_RNAs_2 | 152533431 | 153088 | 996.38 | 1792 |
| Mid_RNAs_3 | 158380257 | 160107 | 989.22 | 1799 |
| Acr_RNAs_1 | 121337254 | 116221 | 1044.02 | 1785 |
| Acr_RNAs_2 | 110923585 | 110192 | 1006.64 | 1749 |
| Acr_RNAs_3 | 118226939 | 115259 | 1025.75 | 1781 |

Note: Ros, WY, San, GHZ, Pin, Son, Mid, and Acr are abbreviations of Anthurium cultivars Rosa, Wu Yang, Sante, Guang Hua Zi Yun, Pink Champion, Sonate, Midori, and Acropolis, respectively. RNAs 1, 2, and 3 indicate three biological replicates.

**TABLE S3** The result of unigene assembly.

| Sample | Total Length | Total number | Average Length | N50 Length |
| --- | --- | --- | --- | --- |
| Unigene | 126410232 | 62013 | 2038.45 | 2519 |

**TABLE S4** A list of primers used in this study for qRT-PCR analysisy.

| Gene | Unigene | Primers | Sequence (5′–3′) |
| --- | --- | --- | --- |
| *AaMYB2* | Unigene32682 | MYB2-F | AACTACGTGAAGCCCACCATC |
|  |  | MYB2-R | ACCAGCTATGAGAGACCACCG |
| *AaMYB124* | Unigene12793 | MYB124-U1F | GCTGCCAACAAGTAGCCATT |
|  |  | MYB124-U1R | GGAGTCATTGCTGCTATGCC |
| *AaLAR* | Unigene16430 | LAR-F | CCGCCATGACCGTGAACTTCC |
|  |  | LAR-R | CCTACATCTGCTGCAACTCCATCG |
| *AaANR* | Unigene7781 | ANR-F | TTCAGCGTCAGGTTAGCAC |
|  |  | ANR-R | CCACAAGCCCGAGTTTATT |
| *AaHemB* | Unigene51685 | HemB-F | AGTCTCCACAGCACTCAGTC |
|  |  | HemB-R | TGCAGGTGATAGACGGGTTT |
| *AaPor* | Unigene38255 | Por-U1F | GCCAGATTCTTCCTCGGAGA |
|  |  | Por-U1R | TCCTCGACGACCTCAACAAA |
| *AaGADPH* |  | GADPH-F | TTTGGGTGACAGCAGGTCGAG |
|  |  | GADPH-R | AAGCCACCACTGCCAACCG |


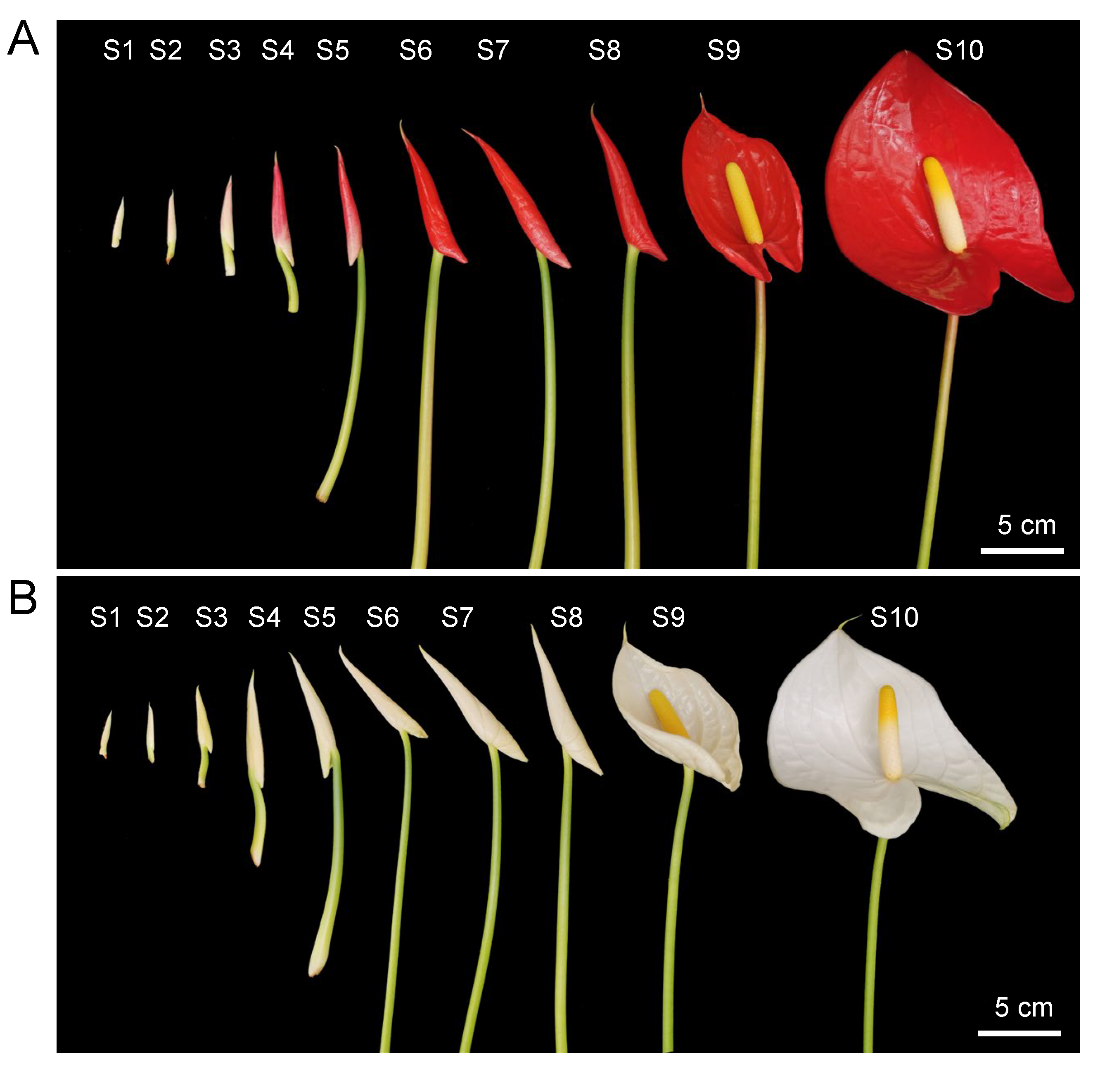


**FIGURE S1**. Spathe developmental stages from 1 to 10 in red spathe cultivar Te Lun Sa (**A**) (TLS) and its mutant cultivar, white spathe (**B**). Stage 1, spathe has not emerged; stage 2, spathe is first visible; stage 3, spathe protrudes from the protecting leaf sheath; stage 4, spathe tightly furled but peduncle starts to elongate; stage 5, spathe remains furled but peduncle elongate to about 10-15 cm; stage 6, spathe has almost coloration but remains furled with slight expansion, peduncle elongate to about 20-25 cm; stage 7, spathe shows full coloration without being open and peduncle is 30-35 cm; stage 8, similar to stage 7, but spathe shows more expansion and peduncle elongate to 40-45 cm; stage 9, spathe is unfolded; and stage 10, spathe fully open without dehisced. Note: This classification was modified based on that of Collette (2004).


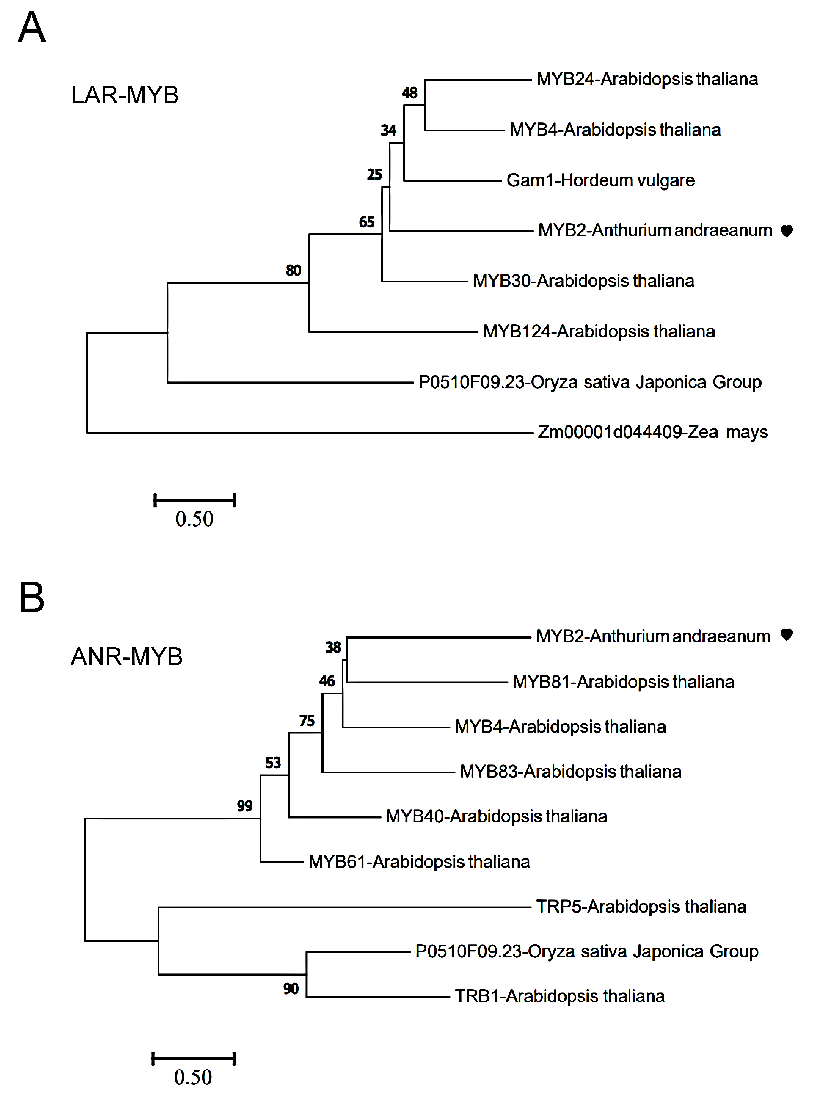


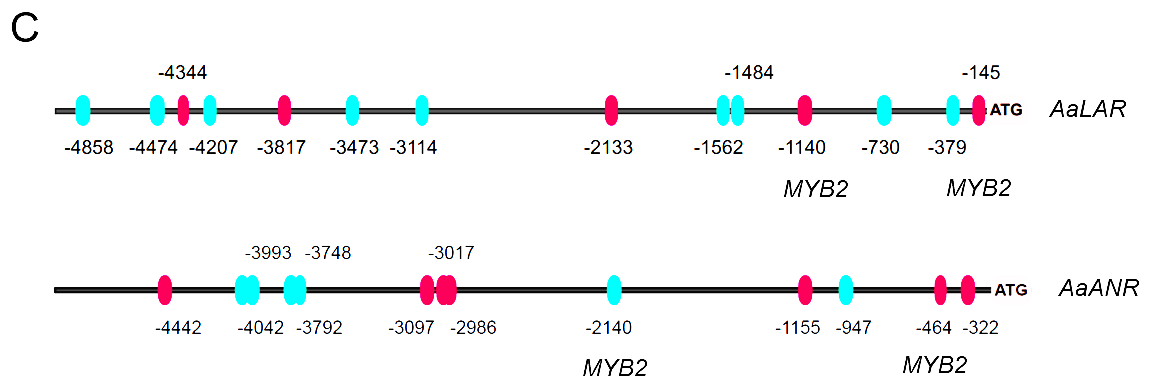


**FIGURE S2**. Phylogenetic analyses of putative homologs of *AaMYB2* with putative binding motifs on *AaLAR* (**A**) and *AaANR* (**B**) promoters, respectively. ClustalW multiple sequence alignment was performed with default parameters in MEGA 7.0.26 and the phylogenetic tree was constructed using the neighbor-joining method. Evolutionary distances were calculated using the *P* distance method; scale bar indicates amino acid substitutions; bootstrap values from 1,000 replicates were presented as percentages shown next to the branches. (**C**) Prediction of MYB transcription factor binding sites in the promoters of *AaLAR* and *AaANR*. The binding sites were shown as red ovals (positive strand) and cyan ovals (negative strand).


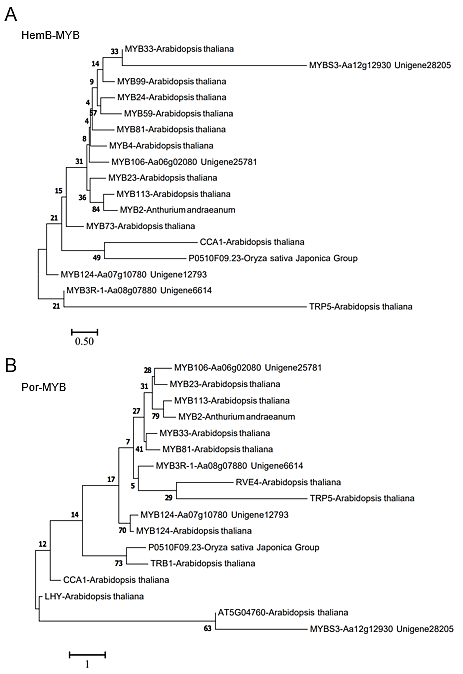


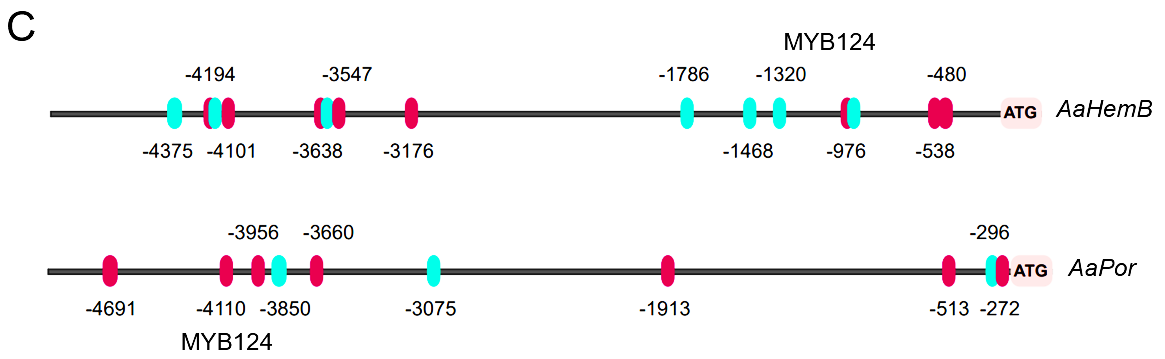


**FIGURE S3**. Phylogenetic analyses of putative homologs of and *AaMYB124* with putative binding motifs on *AaHemB* (**A**) and *AaPor* (**B**) promoters, respectively. ClustalW multiple sequence alignment was performed with default parameters in MEGA 7 and the phylogenetic tree was constructed using the neighbor-joining method. Evolutionary distances were calculated using the *P* distance method; scale bar indicates amino acid substitutions; bootstrap values from 1,000 replicates were presented as percentages shown next to the branches. (**C**) Prediction of MYB transcription factor binding sites in the promoters of *AaHemB* and *AaPor*. The binding sites were shown as red ovals (positive strand) and cyan ovals (negative strand).


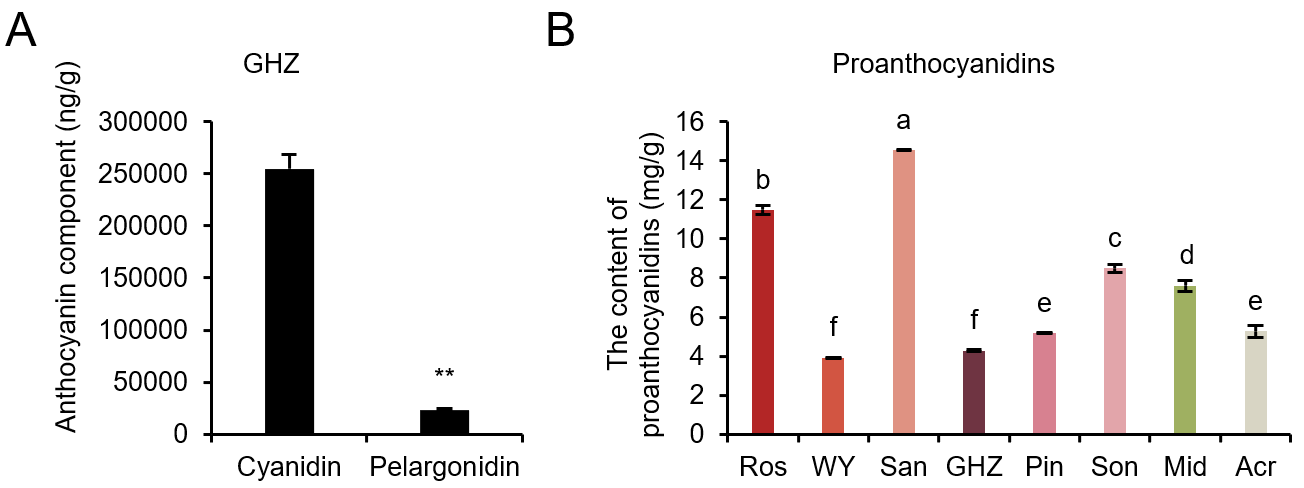


**FIGURE S4.** Anthocyanin component and proathocyanidin content in spathes of Anthurium cultivars of Ros, WY, San, GHZ, Pin, Son, Mid, and Acr, which are Rosa, Wu Yang, Sante, Guang Hua Zi Yun, Pink Champion, Sonate, Midori, and Acropolis, respectively. (**A**) Main anthocyanin compounds in GHZ. (**B**) The content of proathocyanidins in the eight cultivars. The values are means with corresponding standard error (n = 5). Asterisk (**) indicates significant differences analyzed by *t* test at *P* < 0.01 level. Different letters above bars indicate significant differences analyzed by Fisher’s LSD test at *P* < 0.05 level.


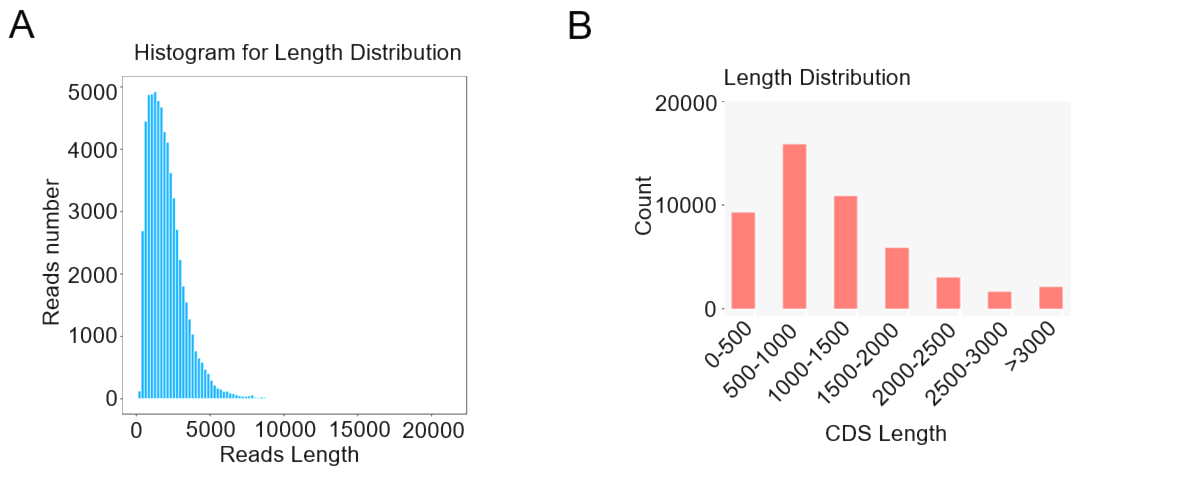


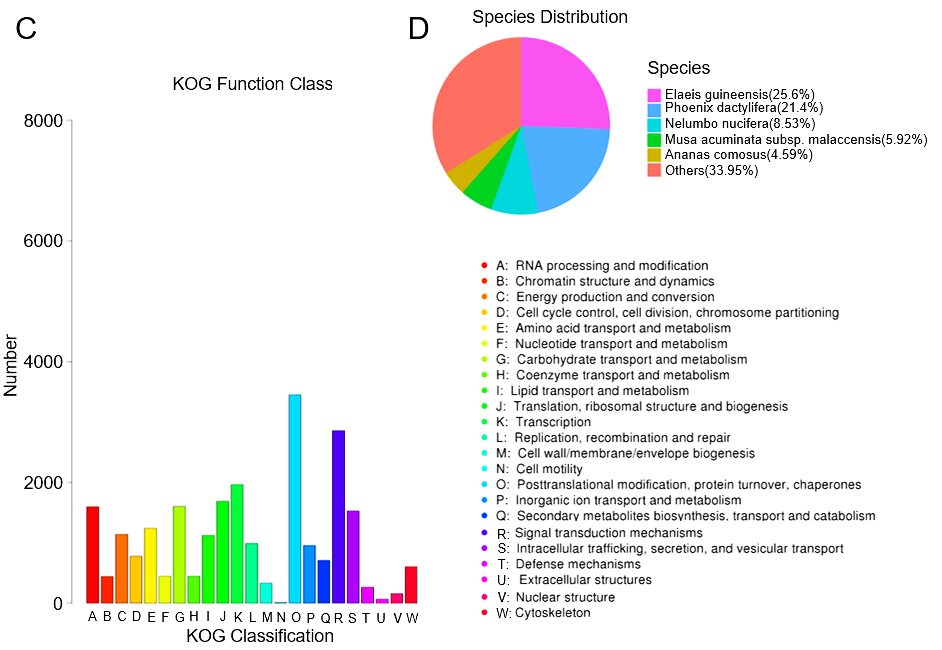


**FIGURE S5.** Analysis of RNA-Seq data of *A. andraeanum.* (**A**) The length distribution of unigenes. (**B**) Length distribution of CDS. (**C**) KOG classification where the x-axis represents KOG categories, and the y-axis represents the number of isoforms. (**D**) The species homology distribution of unigene BLAST hits in the NR database with a cut-off *E* value of 10^-5^ for each sequence.


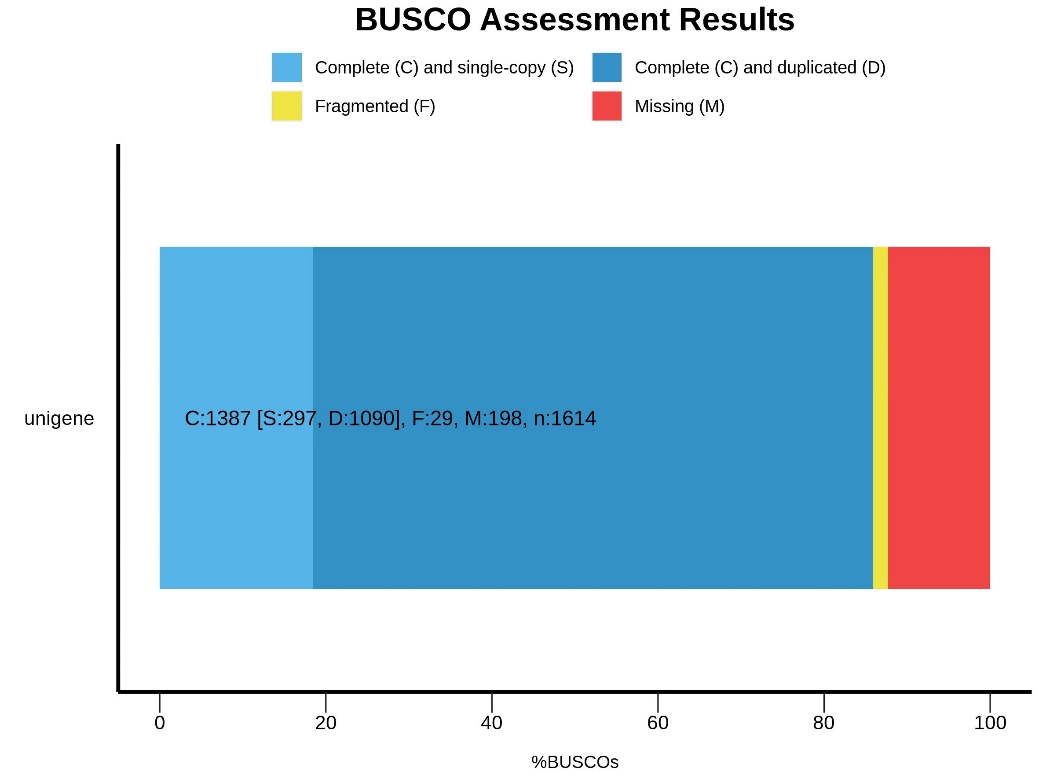


**FIGURE S6.** The completeness analysis of the transcriptome assembly on BUSCO.


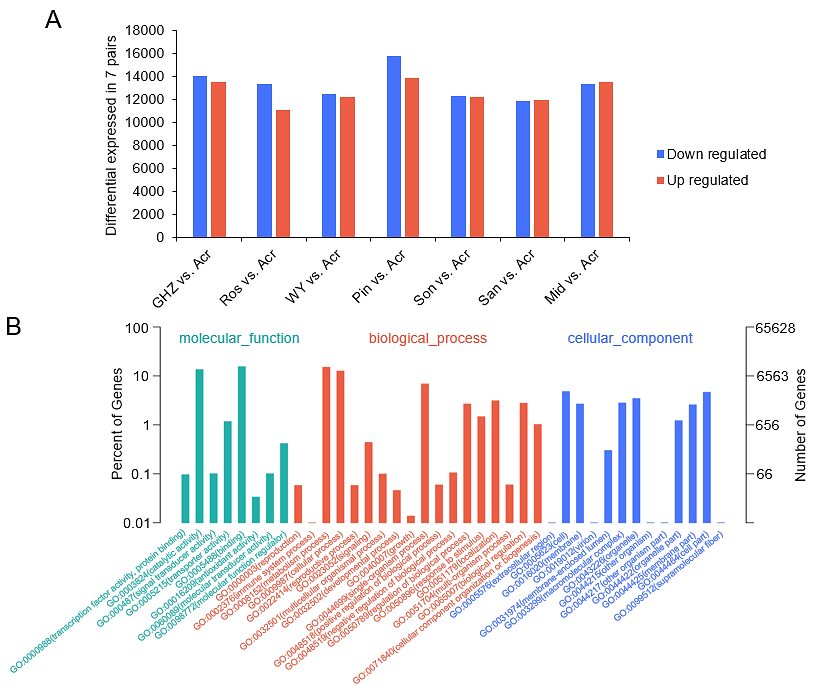


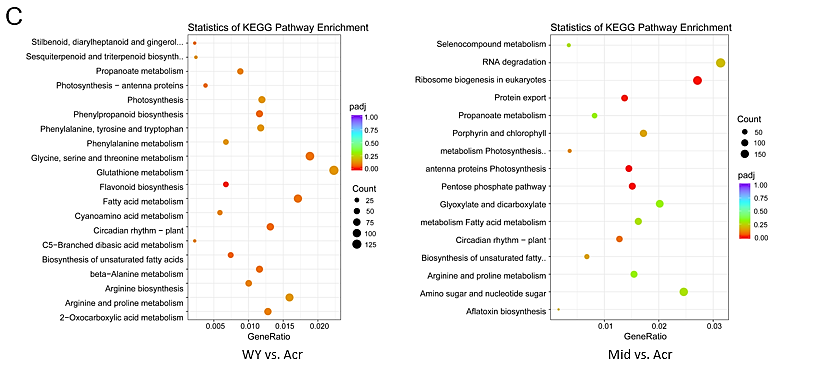


**FIGURE S7.** Statistical analysis of DEGs as well as GO and KEGG enrichment analysis of all DEGs. (**A**) Number of DEGs (padj < 0.05 and | log2(Fold Change) |>1) among all comparison units. (**B**) Go enrichment of all DEGs. The annotated unigenes were clustered under three categories containing molecular function (green bars), biological process (red bars), and cellular component (blue bars). (**C**) KEGG enrichment analysis among WY (Wu Yang) vs. Acr (Acropolis), and Mid (Midori) vs. Acr are shown.


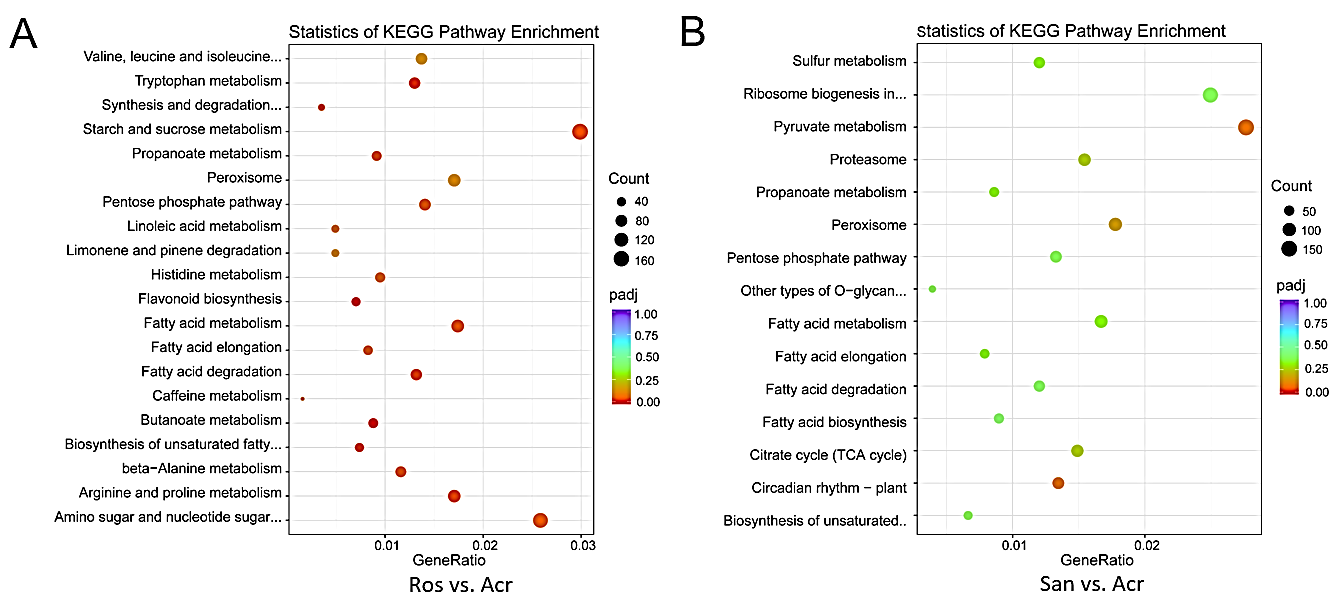


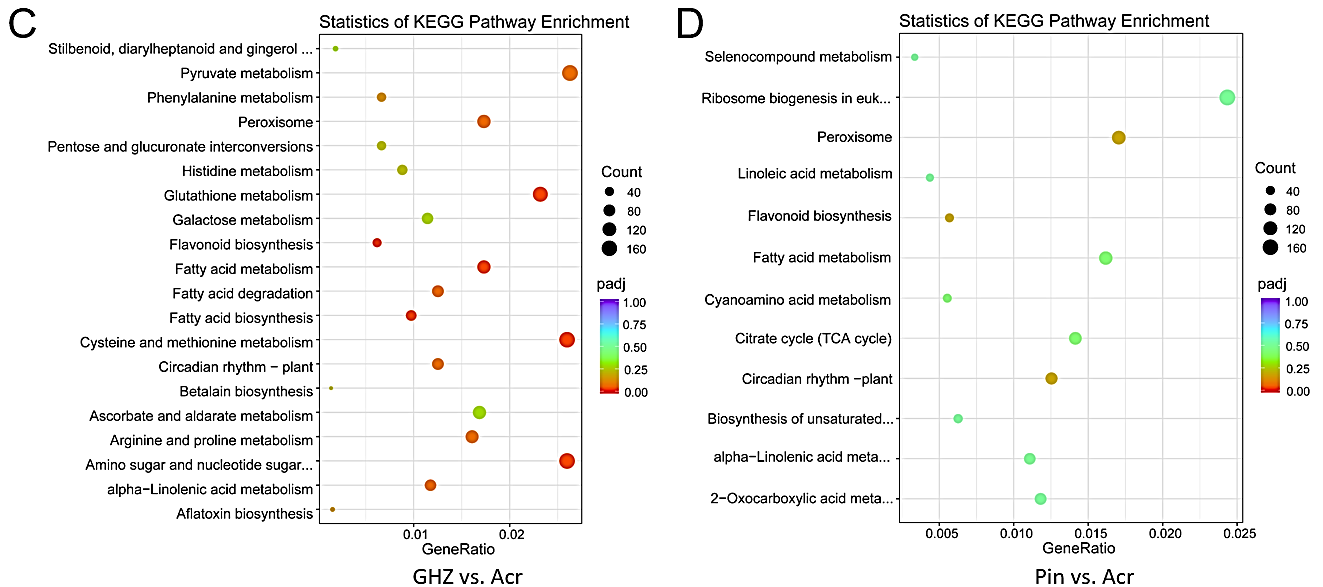


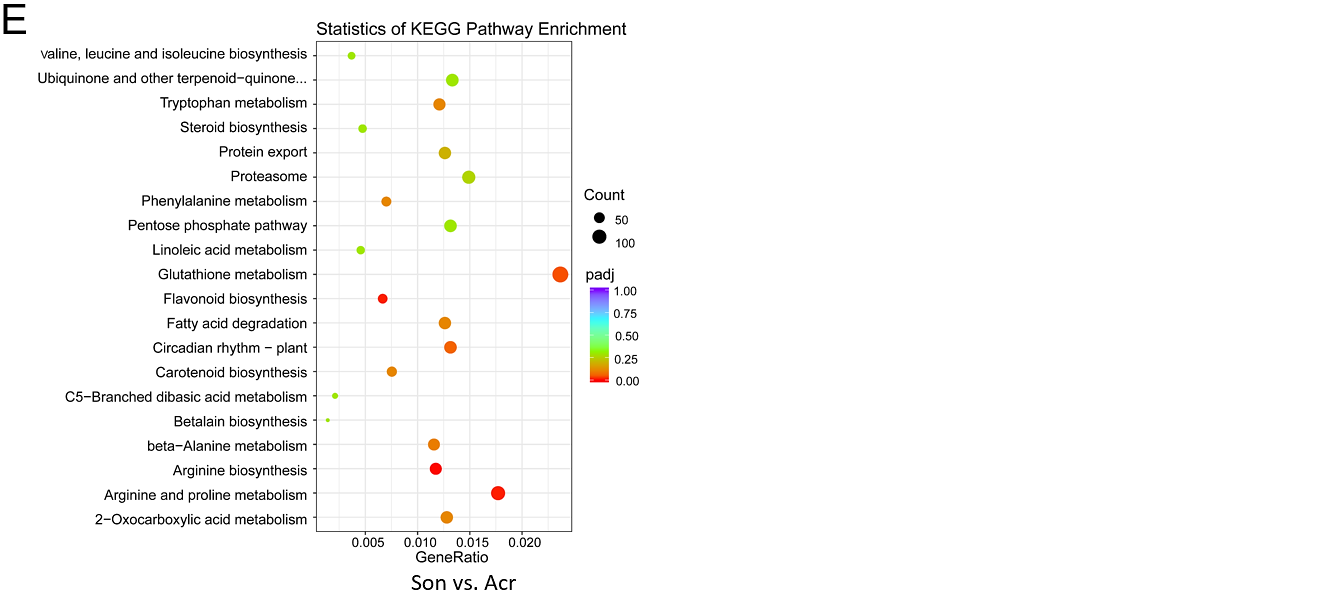


**FIGURE S8.** Statistical analysis of KEGG enrichment analysis of all DEGs. KEGG enrichment analysis among Ros (Rosa) vs. Acr (Acropolis) (**A**); San (Sante) vs. Acr (**B**); GHZ (Guang Hua Zi Yun) vs. Acr (**C**); Pin vs. Acr (**D**); and Son (Sonate) vs. Acr (**E**).


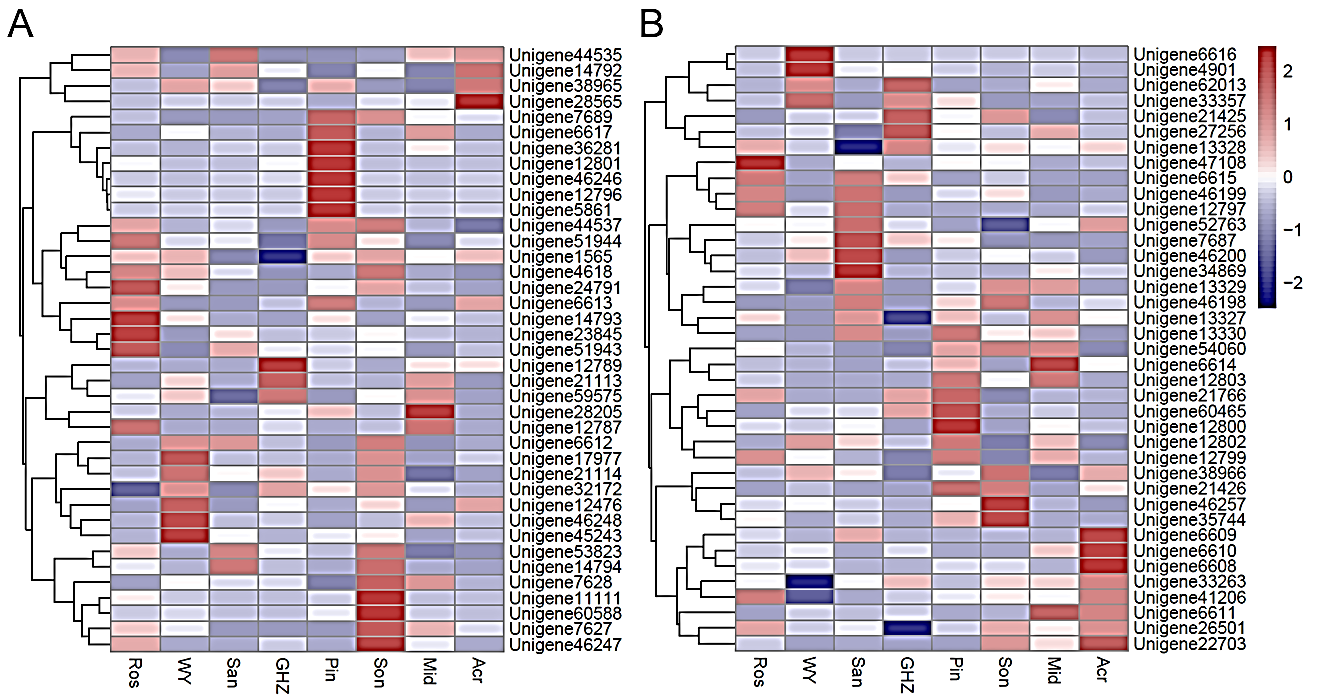


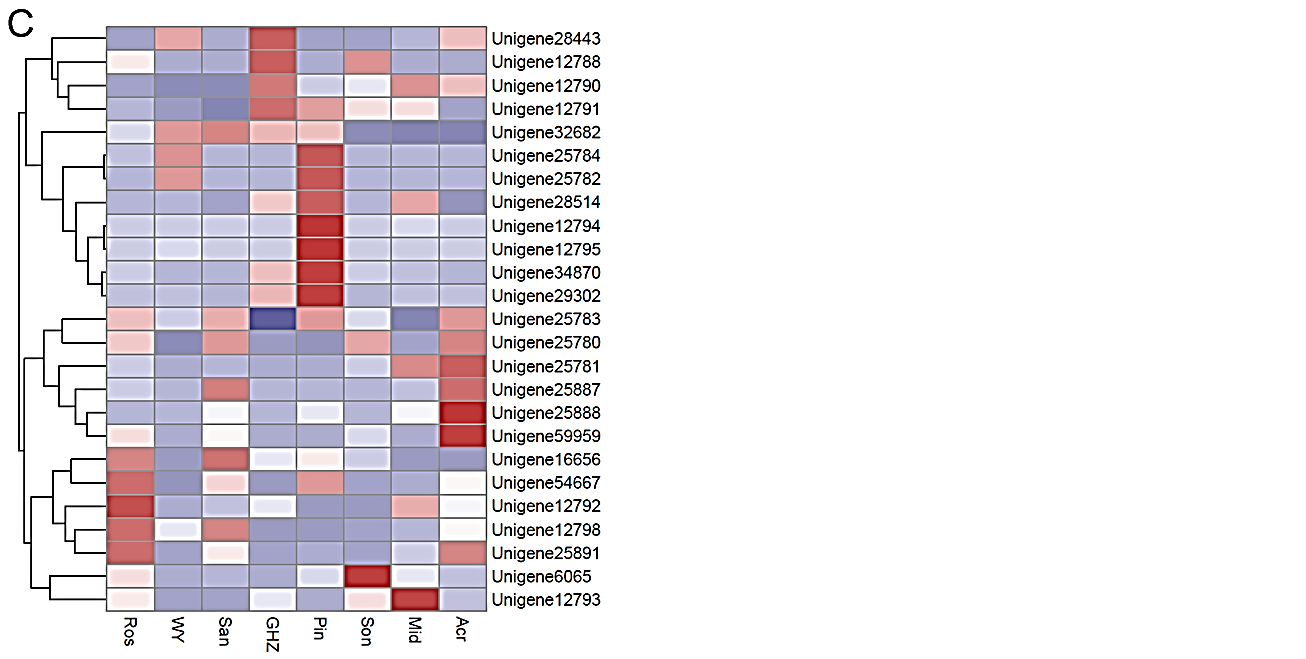


**FIGURE S9**. Heat map clustering and phylogenetic analyses of differentially expressed 103 MYB members in anthurium spathes. (**A**) 39 unigenes of MYB. (**B**) Another 38 unigenes of MYB. (**C**) 26 unigenes of MYB. In the heatmap, purple color represents low adjacency (negative correlation), while red represents high adjacency (positive correlation).


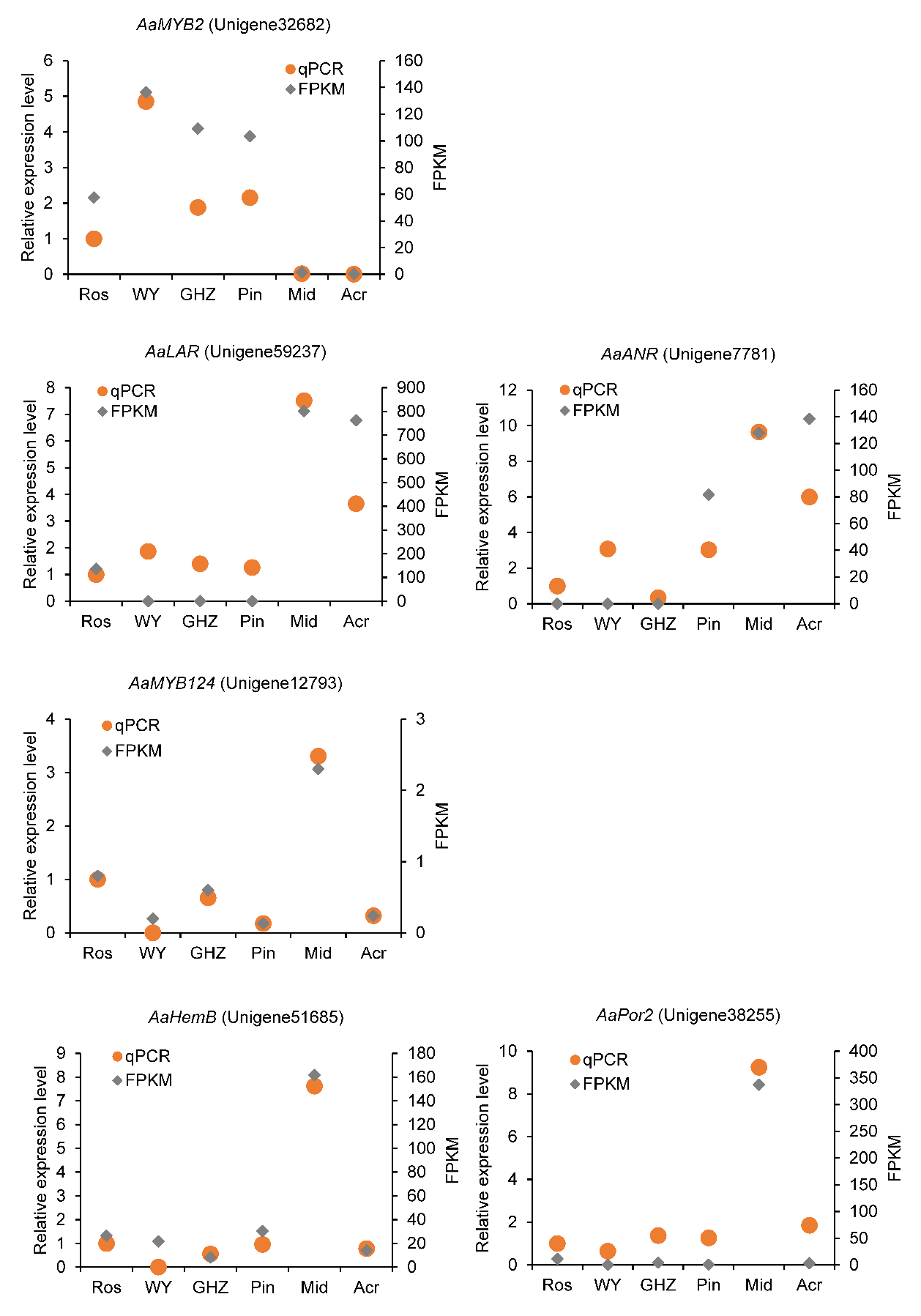


**FIGURE S10**. A comparison of relative expression levels of mentioned genes between qRT-PCR results and the RNA-Seq results. Expression analysis of *AaMYB2*, *AaLAR*, *AaANR*, *AaMYB124*, *AaHemB*, and *AaPor* in the spathe tissue of six cultivars. Expression levels were normalized to the expression of the *GADPH* gene. Data are presented as means of three biological replicates.


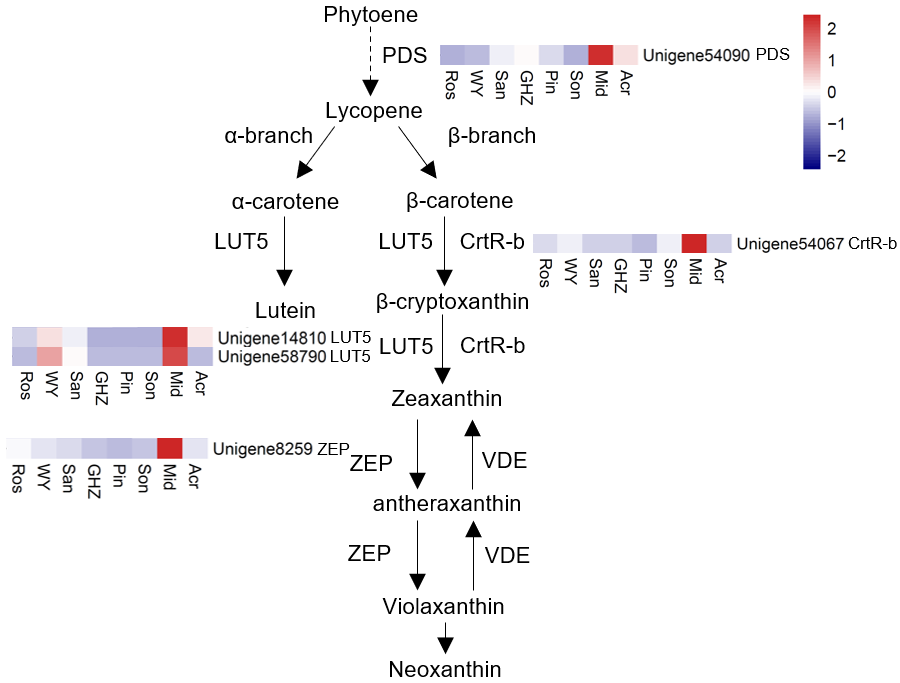


**FIGURE S11.** DEGs involved in the carotenoid biosynthesis and their expression levels. Heatmaps were constructed based on reads per kilobase per million mapped reads (RPKM) of eight anthurium cultivars with different spathe colors. Ros, WY, San, GHZ, Pin, Son, Mid, and Acr represent anthurium cultivars Rosa, Wu Yang, Sante, Guang Hua Zi Yun, Pink Champion, Sonate, Midori, and Acropolis, respectively.

**Supplementary file 1 (File S1): TGICL parameter**

# TGICL Configuration file

#

# Generate HTML reports. Default value 'no'

HTML_REPORT = yes

# The sequence file in fasta format

DB_FILE =

# Quality file

#DB_QUALITY =

# performs more restrictive, layout-based clustering

# instead of simple transitive closure. Default value 'no'

#CLUSTER_LAYOUT = yes

#keep only sequence names with prefix SEQ_FILTER_PREFIX

# SEQ_FILTER_PREFIX =

# skip the mgblast searches (assumed done) but restrict

# further clustering analysis to only the sequences in <seq_list>

#

#SEQ_RESTRICT_LIST =

# pass the ASM_PARAM_FILE as the custom parameter file

# to the assembly program <asmprog.psx>

#ASM_PARAM_FILE =

# A number means to use the specified number of CPUs on

# the local machine. A file name means to use the list of

# PVM nodes given in that file. Default value 1.

#PVM_OR_CPU_LIST =

# user to send email notifications.

# USER_MAIL =

# maximum length of unmatched overhangs. Default value 30.

OVERHANG_LEN = 30

# miminum overlap length. Default value 40.

MIN_OVERLAP = 40

# minimum percent identity for overlaps (PID). Default value 94.

MIN_OVERLAP_PID = 94

# only run the distributed pairwise searches and exit.

# That is no sorting of the pairwise overlaps and no

# clusters generated. Default value 'no'

SEARCHING_ONLY = no

# only run the distributed pairwise searches

# and create the sorted & compressed *_hits.Z file.

# Default value 'no'

SEARCH_AND_SORT = no

# use given CAP3_OPTIONS instead of the default ones

#CAP3_OPTIONS =

# use CLONE_LIST_FILE to put in the same cluster all sequence names

# from the same line.

#CLONE_LIST_FILE =

# ignore lower-case masking in <fasta_db> sequences. Default value 'no'

IGNORE_MASKING = no

# store gap information for all pairwise alignments. Default value 'no'

STORE_GAP_INFO = no

# do not perform all-vs-all search, but search DB_FILE against

# TARGET_DB_FILE instead. Stops after the pairwise hits are generated.

# TARGET_DB_FILE =

# use custom script PAIRWISE_SCRIPT for the distributed

# pairwise searches. Default value 'tgicl_cluster.psx'

#PAIRWISE_SCRIPT =

# use custom script ASM_SCRIPT as the slice assembly script

# Default value 'tgicl_asm.psx'.

#ASM_SCRIPT =

# skip the pairwise searches, only recreate the clusters

# by reprocessing the previously obtained overlaps.

# Default value 'no'

REBUILD_CLUSTERS_ONLY = no

# do not rebuild database indices. Default value 'no'

DB_INDICES_REBUILD = no

# assemble clusters from file CLUSTER_FILE_INPUT

# (do not perform any pairwise clustering)

# CLUSTER_FILE_INPUT =

# number of sequences in a clustering search slice.

# Default value 1000.

MAX_CLUSTER_SEQ = 1000

# (TIGR sequences only) always put in the same cluster all reads

# from the same clone. Default value 'no'

TIGR_CLONE_CLUSTER = no

# attempt to split clusters larger than SEED_MAX_SIZE based on

# seeded clustering. This only works if there are 'et|' or 'np|'

# prefixed entries provided in the input file.

#SEED_MAX_SIZE =

# do not perform assembly, only generate the cluster file

CLUSTER_ONLY = no

# ================== DATABASE SECTION =========================

[DATABASE]

# the user TGICL application use to connect to the database

#USER = tgicl

# password

#PASS = tgiclpass

# database driver. Can be mysql, oracle or postgre

#DRIVER = postgre

#SERVER = localhost

#SCHEMA = tgicldb

**Supplementary file 2 (File S2):**

**Gene annotation using KIPE: MYB annotator and bHLH annotator.**

The flavonoid biosynthesis genes were annotated using KIPEs (https://doi.org/10.3390/plants9091103). The results were as follows,

C4H (Unigene11276) was predicted as C4H.

4CL (Unigene40562) failed to be annotated as 4CL.

CHS (Aa09g08140, Aa09g08170, Aa09g08180, Aa01g20250) was predicted as CHS, but CHS (Aa09g08160) was not.

Only CHI (Unigene23055) was predicted as CHI1, whereas CHI (Unigene23233, Unigene56831, Unigene56832, Unigene20585, Unigene56828) was not annotated.

F3’H (Unigene14530) was predicted as F3H.

F3H (Unigene43097) was not annotated.

DFR (Unigene27457, Unigene4563) was predicted as DFR, while DFR (Unigene9578, Unigene55680, Unigene11713) was not.

5,3GT (Unigene42802, Unigene4645) failed to annotate. There was no 5,3GT in the flavonoid biosynthesis gene database of KIPEs.

ANS (Aa06g11550) was identified as ANS.

All of LAR (Unigene59237, Unigene36956, Unigene61334, Unigene16430, Unigene39856, Unigene39857) were annotated as LAR.

ANR (Unigene7781, Unigene33858) but not ANR (Unigene7779, Unigene27559) were predicted as ANR.

MYB was predicted using the MYB annotator (https://doi.org/10.1186/s12864-022-08452-5). The phylogenetic tree was constructed with representative MYB sequences. The result was as follows,

The phylogenetic relationship of MYB2 (unigene32682) was closest to MYB113, Arabidopsis-thaliana-At1g66370-At2R-MYB113.

The phylogenetic relationship of MYB124 (Unigene12793) was closest to MYB042, Solanum-lycopersicum-v2.3-Solyc05g007160.2-Sl2R-MYB042.

The phylogenetic relationship of MYB106 (Unigene25781) was closest to MYB043, Solanum-lycopersicum-v2.3-Solyc05g007690.1-Sl2R-MYB043.

Some of the assignments are surprising but might be caused by the incomplete transcriptome assembly. C4H and 4CL are probably not relevant for the anthocyanins. MYB2 (unigene32682) seems to be the important anthocyanin regulator and is thus a promising candidate.

bHLH was predicted using the bHLH annotator (https://github.com/bpucker/bHLH_annotator). The phylogenetic tree was constructed with representative bHLH sequences. The results were as follows:

The phylogenetic relationship of bHLH35 (Unigene4957) was closest to AtbHLH027/AtbHLH035 of Arabidopsis thaliana.

The phylogenetic relationship of bHLH51 (Unigene33982) was closest to AtbHLH051 of Arabidopsis thaliana.

The phylogenetic relationship of bHLH1 (Aa14g06660) was closest to AtbHLH042 of Arabidopsis thaliana.

The phylogenetic relationship of bHLH113 (Aa14g02910) was closest to AtbHLH123 of Arabidopsis thaliana.

The phylogenetic relationship of bHLH62 (Unigene57010) was closest to AtbHLH062 of Arabidopsis thaliana.

The phylogenetic relationship of bHLH49 (Unigene58607) was closest to AtbHLH137 of Arabidopsis thaliana.

bHLH57 (Unigene26120) was not annotated.
